# *In-silico* and *in-vitro* functional validation of imidazole derivatives as potential sirtuin inhibitor

**DOI:** 10.3389/fmed.2023.1282820

**Published:** 2023-11-07

**Authors:** Uma Maheswara Rao Dindi, Suhadha Parveen Sadiq, Sameer Al-Ghamdi, Naif Abdurhman Alrudian, Salman Bin Dayel, Abdulwahab Ali Abuderman, Mohammad Shahid, Thiyagarajan Ramesh, Ravikumar Vilwanathan

**Affiliations:** ^1^Cancer Biology Laboratory, Department of Biochemistry, School of Life Sciences, Bharathidasan University, Tiruchirappalli, Tamil Nadu, India; ^2^Department of Family and Community Medicine, College of Medicine, Prince Sattam Bin Abdulaziz University, Al-Kharj, Saudi Arabia; ^3^Dermatology Unit, Internal Medicine Department, College of Medicine, Prince Sattam Bin Abdulaziz University, Al-Kharj, Saudi Arabia; ^4^Department of Basic Medical Sciences, College of Medicine, Prince Sattam Bin Abdulaziz University, Al-Kharj, Saudi Arabia

**Keywords:** imidazole, HDAC, inhibitors, epigenetics, sirtuins

## Abstract

**Introduction:**

Epigenetic enzymes can interact with a wide range of genes that actively participate in the progression or repression of a diseased condition, as they are involved in maintaining cellular homeostasis. Sirtuins are a family of Class III epigenetic modifying enzymes that regulate cellular processes by removing acetyl groups from proteins. They rely on NAD^+^ as a coenzyme in contrast to classical histone deacetylases (HDACs) (Class I, II, and IV) that depend on Zn^+^ for their activation, linking their function to cellular energy levels. There are seven mammalian sirtuin isoforms (Sirt1-7), each located in different subcellular compartments. Sirtuins have emerged as a promising target, given that inhibitors of natural and synthetic sources are highly warranted. Imidazole derivatives are often investigated as sirtuin regulators due to their ability to interact with the binding site and modulate their activity. Imidazole bestows many possible substitutions on its ring and neighboring atoms to design and synthesize derivatives with specific target selectivity and improved pharmacokinetic properties, optimizing drug development.

**Materials and methods:**

Ligand preparation, protein preparation, molecular docking, molecular dynamics, density function theory (DFT) analysis, and absorption, distribution, metabolism, and excretion (ADME) analysis were performed to understand the interacting potential and effective stability of the ligand with the protein. RT-PCR and Western blot analyses were performed to understand the impact of ligands on the gene and protein expression of Class III HDAC enzymes.

**Results and discussion:**

We evaluated the sirtuin inhibition activity of our in-house compound comprised of imidazole derivatives by docking the molecules with the protein data bank. ADME properties of all the compounds used in the study were evaluated, and it was found that all fall within the favorable range of being a potential drug. The molecule with the highest docking score was analyzed using DFT, and the specific compound was used to treat the non-small cell lung cancer (NSCLC) cell lines A549 and NCI-H460. The gene and protein expression data support the *in-silico* finding that the compound Ethyl 2-[5-(4-chlorophenyl)-2-methyl-1-H-Imidazole-4-yl) acetate has an inhibitory effect on nuclear sirtuins. In conclusion, targeting sirtuins is an emerging strategy to combat carcinogenesis. In this study, we establish that Ethyl 2-[5-(4-chlorophenyl)-2-methyl-1-H-Imidazole-4-yl) acetate possesses a strong inhibitory effect on nuclear sirtuins in NSCLC cell lines.

## Highlights

- Ethyl 2-[5-(4-chlorophenyl)-2-methyl-1-H-Imidazole-4-yl) acetate has a higher docking score, glide score, and glide energy compared to other compounds within the sirtuin family proteins.
- Ethyl 2-[5-(4-chlorophenyl)-2-methyl-1-H-Imidazole-4-yl) acetate has better stability when interacting with nuclear sirtuins.
- Imidazole derivative effectively reduces the viability of cancer cell lines A549 and NCI-H460 at lower concentrations compared to imidazole.
- Ethyl 2-[5-(4-chlorophenyl)-2-methyl-1-H-Imidazole-4-yl) acetate greatly affects sirtuins family members' gene expression and protein expression.

## 1. Introduction

Imidazole derivatives have shown promising potential in combating various types of cancers. They can be designed and synthesized to specifically target molecular pathways and proteins that are crucial for the growth and survival of cancer cells (1, 2). This targeted approach minimizes damage to healthy cells and tissues, leading to fewer side effects. Imidazole derivatives have demonstrated the ability to inhibit cell proliferation and potentially induce apoptosis in breast cancer cells (3). They can inhibit angiogenesis, and by cutting off the blood supply to the tumor, these compounds can deprive the cancer cells of essential nutrients, thereby hindering their growth (4). Imidazole derivatives can sensitize cancer cells to the effects of chemotherapy and radiation therapy (5). They have demonstrated better antimicrobial, antiviral, anti-inflammatory, antifungal, antiparasitic, and anticancer properties compared to other synthetic compounds (6). The significance of imidazole derivatives lies in their diverse biological activities, structural versatility, and potential therapeutic applications. Using imidazole derivatives to study epigenetic enzymes to modulate their activity and restore normal gene expression patterns is an active area of research (7). Imidazole and its derivatives can either work as a friend or foe for epigenetic enzymes. Our previous research emphasized that heterocyclic imidazole derivatives intervene in classic histone deacetylase (HDAC) enzyme activity and inhibit their operations in lung cancer cell lines (8). However, class III-HDAC is the most active form in orchestrating the progression and suppression of various diseases (9). Furthermore, imidazole derivatives can function as activators or inhibitors of sirtuins. As activators, imidazole derivatives can typically bind to the protein and increase their enzymatic activity, leading to enhanced deacetylation or ADP-ribosylation of target proteins. On the other hand, as inhibitors, they can block sirtuin activity, leading to altered gene expression patterns and cellular functions. Sirtuins play a crucial role in metabolic pathways, including glucose and lipid metabolism (10). Activation of sirtuins by imidazole derivatives or other compounds may enhance metabolic efficiency and potentially offer therapeutic benefits in addressing metabolic disorders (11). Sirtuins have also been implicated in neuroprotection and cognitive function. Therefore, imidazole derivatives that modulate sirtuin activity may have potential applications in neurodegenerative diseases, such as Alzheimer's and Parkinson's, by promoting neuronal survival and enhancing cellular stress responses (12). Sirtuins can act as tumor suppressors by regulating cell cycle progression, DNA repair, and apoptosis (13). Sirtuin activators, including certain imidazole derivatives, may have anticancer properties by promoting tumor cell death and inhibiting tumor growth. Furthermore, based on the type of cancer tissues, the activity of sirtuins differs (14), making the development of effective and selective sirtuin regulators a complex task.

The development of sirtuin inhibitors as potential therapeutic agents for combating cancer, including lung cancer, is an active area of research (15). Inhibiting specific sirtuin isoforms may offer opportunities for novel cancer treatments (16). There are seven mammalian sirtuin isoforms (Sirt 1-7), each with unique functions and subcellular localizations (17). To develop effective inhibitors, it is crucial to understand the roles of individual sirtuin isoforms in lung cancer biology and identify those most relevant to disease progression. As sirtuins actively participate in cancer progression and regulation (18, 19), designing imidazole derivatives that can interfere with the activity of sirtuin has become imperative. Structure-based drug design and screening approaches can be used to identify small molecules with potential inhibitory activity against sirtuin isoforms. Imidazole derivatives and other chemical scaffolds can be modified and optimized to interact with the NAD^+^-binding site of the sirtuin isoform. Achieving isoform specificity is critical in the development of sirtuin regulators to avoid unwanted effects on non-target sirtuin isoforms, which may have distinct roles in normal cellular functions. Among the top 75% of the small-molecule medications containing heterocycles with nitrogen, imidazole ranks within the top 10, favoring a ring system that acts as essential building blocks for the development of novel drugs (20). In this context, multiple derivatives of imidazole were synthesized to study their characteristics with respect to cancer (21). Given that the action of imidazole derivatives on the modulation of sirtuins could have a beneficial effect in transformed cells, in the present study, we aimed to understand the inhibitory effect of our in-house imidazole derivatives on sirtuins.

## 2. Materials and methods

### 2.1. Ligand preparation

**Figure 1:**
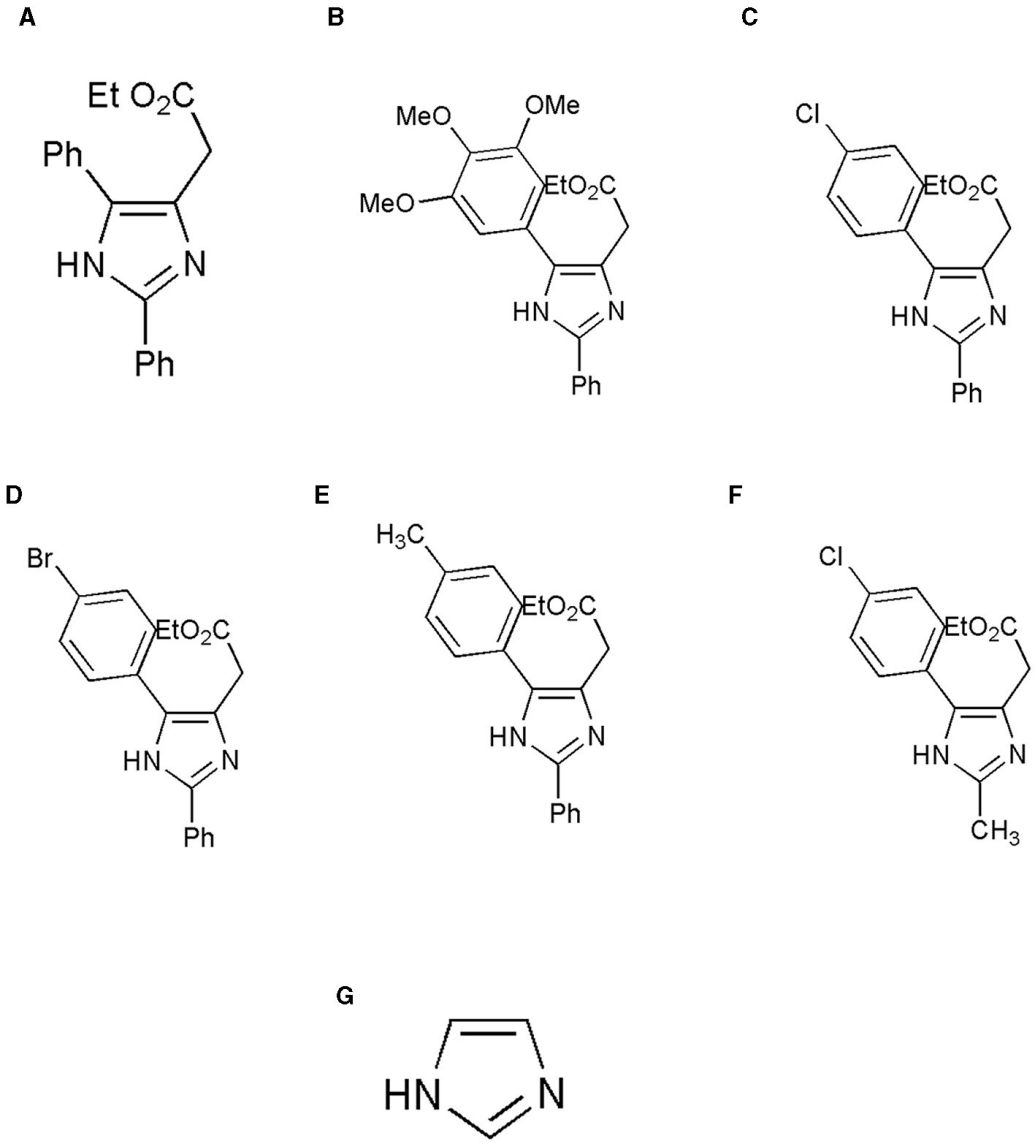
Structure of the ligands generated using ChemDraw is shown. **(A)** Ethyl 2-(2,5-diphenyl-1H-imidazole-4-yl) acetate, **(B)** Ethyl 2-[2-phenyl-5-(3,4,5-trimethoxyphenyl)-1H-imidazol-4-yl] acetate, **(C)** Ethyl 2-[5-(4-chlorophenyl)-2-phenyl-1H-imidazol-4-yl] acetate, **(D)** Ethyl 2-[5-(4-bromophenyl)-2-phenyl-1H-imidazol-4-yl] acetate, **(E)** Ethyl 2-{2-phenyl-5-[4-(trifluoromethyl) phenyl]-1H-imidazol-4-yl} acetate, **(F)** Ethyl 2-[5-(4-chlorophenyl)-2-methyl-1-H-Imidazole-4-yl) acetate, and **(G)** Imidazole. **(A)** Adapted from (8) with permission from Elsevier. Copyright ©2020 Elsevier. **(F)** Adapted with permission from (22). Copyright ©2014 American Chemical Society.

An imidazole and six imidazole derivatives were generated in the ligand library. ChemDraw Pro 8.0 was used to draw the 3D structures of the ligands (Figure 1). The ligand structures were optimized using Maestro version 13.2.128, Release 2022-2, and the LigPrep module was performed with force field OPLS_2005.

### 2.2. Protein preparation and receptor grid generation

Barring Sirt4, the remaining six sirtuin isoforms were considered in the *in-silico* study. The protein data bank (PDB) structures of Sirt1, Sirt2, Sirt3, Sirt5, Sirt6, and Sirt7 were recovered from the Research Collaboratory for Structural Bioinformatics (RCSB) as 4I5I, 4RMH, 4JSR, 6LJK, 3K35, and 5IQZ, respectively. The proteins subjected to docking studies were pre-processed, optimized, removed water, and minimized using a protein preparation wizard. Water molecules, non-essential atoms, and attached ligands were annihilated. Further preparation was performed by adding missing atoms in the protein residues, fixing the alternate confirmations, and adding hydrogen. Grid generation surrounding the binding site was achieved as per the protocol followed in Maestro version 13.2.128, Release 2022-2.

### 2.3. Glide docking

Grid-based Ligand Docking with Energetics (GLIDE) Maestro version 13.2.128, Release 2022-2, was used to dock the desired imidazole derivatives with all six PDB structures. The ligand molecules were ranked based on their interactive score and energy in order to understand their interaction with protein molecules. Sirt 1-7 proteins, except Sirt4, were docked against the imidazole derivatives. Correspondence to the docking score, glide score, and glide energy of the ligand and the protein, poses were visualized using Maestro GUI.

### 2.4. Molecular dynamic simulation using desmond

Based on the docking score, the top ligand bound to all the sirtuins was subjected to molecular dynamic (MD) simulation using Desmond program version 7.0 (academic version). An orthorhombic periodic box of dimension 10 Å3 with solvent TIP3P at force field OPLS_2005 was set, and the neutralization of the system at stable pH was carried out by adding counter ions (Na+ and Cl–) to the system builder. The protein-ligand complex underwent minimization of energy and attained a pre-equilibrium state. Molecular dynamics simulations were conducted for a duration of 100 ns, utilizing a relaxation time of 1 ps and maintaining a consistent temperature of 300 kelvin. Throughout the simulation, a series of 1000 frames was generated using a time step of 20 ps, serving as the foundation for deriving average structures during the production phase. Additionally, the root mean square fluctuation (RMSF) and root mean square deviation (RMSD) were independently assessed for the protein-ligand complex structure and the protein structure. The purpose of this simulation was to investigate the dynamic stability of the complexes and was visualized by plotting RMSF and RMSD values against time.

### 2.5. DFT analysis

The electronic structural characteristics play a pivotal role in elucidating the molecular interactions governing small molecule dynamics, molecular structure, and quantum properties. To delve into this, the Gaussian 09 software package was employed to conduct density functional theory (DFT) calculations on the primary ligand. Using the B3LYP-D3 hybrid functional and the 6-311G^**^++ (2d, 2p) basis set, the calculation encompassed the determination of the highest occupied molecular orbital (HOMO) and lowest unoccupied molecular orbital (LUMO) energy levels, collectively known as frontier orbitals. The HOMO represents the outermost electron of the ligand capable of donation upon binding to a protein, while the LUMO represents the region of the ligand with an affinity to accept electrons from the protein during complex formation. As an essential metric, the energy gap (HOMO-LUMO) was computed to clarify chemical potential, electron affinity, chemical stability, hardness, and the chemical potential of the ligand molecules.

### 2.6. ADME analysis

The imidazole structure was constructed using Chemdraw, and various interactable R groups were added to the side chains. The structures were then exported into.sdf files and read in Schrodinger Maestro software version 13.2.128(2022-2). The structures were analyzed using the Qikprop module to understand their chemical properties. All the compounds underwent absorption, distribution, metabolism, and excretion (ADME) analysis where the molecular weight, number of hydrogen bonds accepted and donated, solvent accessible surface area (SASA), force-field overlapping scoring algorithm (FOSA), flexible inhibitor surface anchoring (FISA), percentage of human absorption, and the permeability to the gut and brain were calculated. This analysis indicated the ability of the domestic compounds to acquire the qualities of being a promiscuous drug.

### 2.7. Cell culture maintenance

The A549 (adenocarcinoma) and NCI-H460 (large cell carcinoma) human non-small-cell lung cancer cell lines used in this study were purchased from the National Center for Cell Science (NCCS) Pune, India. The reagents for cell culture, such as Dulbecco's modified eagle's medium (DMEM), fetal bovine serum (FBS), 10 × phosphate-buffered saline (pH 7.2), 10 × Trypsin-EDTA, and 100 × penicillin-streptomycin (pen strep, pH 7.2) antibiotic solution were from Hi-Media Laboratories, Mumbai, India. Cell culture plates were purchased from Wuxi NEST Biotechnology Co. Ltd., Jiangsu, China. The other plastic wares were purchased from Tarsons Pvt. Ltd.. The complete medium, which included DMEM, 10% FBS, and 1% pen strep solution, was used for culturing the cells. The cultured cell lines were maintained in a CO_2_ incubator at an atmospheric temperature of 37°C and supplied with 5% CO_2_.

### 2.8. Cell viability assay by MTT

The half maximal inhibitory concentration (IC_50_) is a quantitative measure of a substance (e.g., drug) to inhibit biological processes or biological components by 50%. The MTT assay is a well-known assay to determine IC_50_. The cell lines grown in monolayer were trypsinized and pelleted. The cell pellet was dissolved in a complete medium. The cells were then counted using the Trypan blue and hemacytometer. Approximately 1 × 10^4^ cells were seeded in 96-well plates and incubated overnight in a CO_2_ incubator. After incubation, the old media were removed, and serum-free media were added to the wells. Imidazole and its derivative Ethyl 2-[5-(4-chlorophenyl)-2-methyl-1-H-Imidazole-4-yl) acetate was synthesized as reported earlier (22) and were used to treat the cells seeded with different concentrations. Treated concentrations of imidazole ranged from 100 μM to 1000 μM, and its derivative Ethyl 2-[5-(4-chlorophenyl)-2-methyl-1-H-Imidazole-4-yl) acetate concentration ranged from 50 μM to 500 μM and incubated for 24 h. After 24 h of treatment, 20 μL of 5 mg/mL concentration of MTT (3 (4,5-Dimethyl-2-thiazolyl)-2,5-diphenyl-2H-tetrazolium bromide) was added to the wells, and incubated for 4 h. Later, the media were removed from the wells and 200 μL of dimethyl sulphoxide (DMSO) was added to dissolve the formazan crystals. After 30 min of incubation, the intensity of the purple color formed was measured by an ELISA plate reader (Bio-Rad Laboratories, Hercules, CA, USA) at 595 nm. The higher the intensity, the greater the number of viable cells, and the lower the intensity, the lesser the number of viable cells. MTT (RM1131) and DMSO (GRM5856) were purchased from Hi-Media Laboratories, Mumbai, India. The percentage of viable cells was calculated using the following formula:

% Cell viability = 100 – [(Mean OD of control cells – Mean OD of treated cells)/Mean OD of control cells × 100%]

### 2.9. Quantitative real-time PCR (qrt-PCR)

The non-small cell lung cancer (NSCLC) cell lines A549 and NCI-H460 cells were seeded into culture plates using the complete medium. When the culture reached 80% confluency, the cells were treated with imidazole derivative Ethyl 2-[5-(4-chlorophenyl)-2-methyl-1-H-Imidazole-4-yl] acetate. The IC_50_ concentration of Ethyl 2-[5-(4-chlorophenyl)-2-methyl-1-H-Imidazole-4-yl] acetate in serum-free medium was used for treatment. The control was maintained in a serum-free medium without Ethyl 2-[5-(4-chlorophenyl)-2-methyl-1-H-Imidazole-4-yl] acetate was used as control. After the treatment period, total RNA was isolated from untreated (control) and Ethyl 2-[5-(4-chlorophenyl)-2-methyl-1-H-Imidazole-4-yl] acetate treated A549 and NCI-H460 cell lines using TRIzol reagent (RNA isoplus). The purity and quantity of the total RNA isolated was determined by BioPhotometer (Eppendorf, Hamburg, Germany). 1 μg of total RNA was taken for cDNA construction using the PrimeScript RT reagent kit following the manufacturer's instructions. Gene expression studies were performed in StepOnePlus real-time PCR system (Applied Biosystems, Thermo Fisher Scientific, MA, USA) using 2 × SYBR green master mix [TB Green Premix Ex Taq II (Tli RNase H Plus)]. The sample preparation was done according to the instructions in the datasheet provided by the manufacturer. Next, 0.5 μL of cDNA was used for each 10 μL reaction. The PCR condition was initiated by denaturation at 94°C for 5 min, followed by 40 cycles of denaturation at 94°C for 30 s, annealing at 55°C – 60°C for 30 s (depending on the specific gene), and melt curve stage conditions set at 95°C for 15 s, 60°C for 60 s, and 95°C for 15 s. Glyceraldehyde 3-phosphate dehydrogenase (GAPDH) was used as an endogenous control. For normalization, the Ct value of the target gene was subtracted from the Ct value of GAPDH, which resulted in ΔCt values. The ΔCt values of the target were then subtracted from the ΔCt value of the control. This gave the ΔΔCt value, which is commonly used to find the mRNA expression. The expression is represented in fold change by 2^(−ΔΔ*Ct*)^. The list of primers used in this study is provided in Table 1. Trizol for total RNA extraction (RNA isoplus, cat. # 9108), PrimeScript RT reagent kit (Perfect Real Time, Cat. # RR037A) for cDNA synthesis, and SYBR green master mix [TB Green^®^ Premix Ex Taq™ II (Tli RNaseH Plus, Cat. #RR820A)] for gene expression studies were purchased from Takara Bio Inc. (Japan).

**Table 1:** List of primers used in the study.

**S. No**	**Gene**	**Primer sequence 5^′^-3^′^**	**Annealing temp**	**Product size**
1	GAPDH	F' P: ATGGGGAAGGTGAAGGTCG	60	107
		R' P: GGGTCATTGATGGCAACAATATC		
2	Sirt1	F' P: ACCCAGCTCACCTTCTTT	57	176
		R' P: CCCAGACTTTCCCACTCT		
3	Sirt2	F' P: CTCCCCTTCCAGCTTAAC	57	175
		R' P: TGACACTCACCCCAAGAC		
4	Sirt3	F' P: GGGAGGGGTACAGTGAGG	58.1	177
		R' P: GGGTGACAGAGCGAGATG		
5	Sirt4	F' P: TGGTCATTGCTGGTTTCC	57	170
		R' P: AGGCAGAGGTTGTGGTGA		
6	Sirt5	F' P: GGCTTTGCTTTCCCTTAC	58.1	171
		R' P: AACCTTGGCGATTAGACC		
7	Sirt6	F' P: TCCCATTGTCTAGCCTCA	58.1	181
		R' P: GATGTCGGTGAATTACGC		
8	Sirt7	F' P: TCGGCTCCTCCCTTCTAC	57	180
		R' P: GGGGGCACTTTAGGAACA		

### 2.10. Western blot analysis

The A549 and NCI-H460 cell lines grown in monolayer were treated with Ethyl 2-[5-(4-chlorophenyl)-2-methyl-1-H-Imidazole-4-yl] acetate in serum-free medium, and untreated cell lines in serum-free medium were used as control samples. After the treatment period, the cells were washed with ice-cold PBS two times. Later, RIPA lysis buffer supplemented with protease and phosphatase inhibitor cocktails was added to the plates and incubated for 30 min in cold conditions. After incubation, the cell lysate was scraped using the cell scraper and collected into 1.5-mL tubes, which were then vortexed for 30 min at different intervals, maintaining cold conditions by keeping the tubes on ice. Later, the cell lysate was centrifugated at 12,000 *g* for 20 min at 4°C in the cooling centrifuge, and the supernatant containing the total protein was collected. The protein samples were quantified by Lowry's method. In total, 50 μg of protein from whole protein samples were used for the Western blot analysis. The 50 μg of whole protein was separated using SDS-PAGE (10% and 12% gels), and the separated proteins were transferred onto the nitrocellulose membrane by wet transfer. The membrane was blocked with 5% skimmed milk for 2 h, after which it was washed with 1 × TBST for 5 min. The washing was done three times. Following the TBST wash, the membrane was incubated overnight with specific primary antibodies at 4°C. Once the overnight incubation was over, the membrane was washed three times with 1 × TBST for 5 min each time. The alkaline phosphatase-conjugated secondary antibody specific to the primary antibody was added and incubated for 4 h at 4°C, after which the membrane was washed three times with 1 × TBST for 5 min each time. The blots were developed with BCIP/NBT chromogenic substrate, and the images were scanned. ImageJ software (National Institutes of Health, Bethesda, MD, USA) was used to measure the intensity of the bands. The intensity of the measured target protein of the control sample was divided by the intensity of the measured βeta Actin of the control. Likewise, the intensity of the target protein of the treated sample was divided by the intensity of βeta-actin of the treated sample. The values obtained after nullifying with βeta-actin were used to represent protein expression in fold change by dividing the intensity of the target protein in the treated sample by that in the control sample. RIPA Lysis Buffer System (sc-24948) for protein isolation was purchased from Santa Cruz (CA, USA). Nitrocellulose Membrane (0.45μm, cat.log1620115) and Precision Plus Protein™ Kaleidoscope™ Prestained Protein Standards (#1610375) were purchased from Bio-Rad Laboratories (Hercules, California, United States). PageRuler Prestained Protein Ladder (cat.log 26616) was purchased from Thermo Fisher Scientific (Waltham, MA, USA). The bovine serum albumin, skimmed milk powder, and all the other fine chemicals used in the SDS and Western blotting were purchased from Sisco Research Laboratories Pvt. Ltd. (SRL, Maharashtra, India). The antibodies used in the study of Sirt 1 (9475), Sirt 2 (12650), Sirt 3 (5490), Sirt 5 (8782), Sirt 6 (12486), and Sirt 7 (5360) came from Cell Signaling Technology, USA. β-actin (MAB8929-SP) was purchased from Novus Biologicals (Briarwood, CO, USA). The secondary antibodies, such as goat anti-mouse IgG H&L (alkaline phosphatase) (ab97020) and goat anti-rabbit IgG H&L (alkaline phosphatase) (ab6722), were purchased from Abcam (Cambridge, UK). 5-bromo-4-chloro-3-indolyl phosphate (BCIP)/nitro blue tetrazolium (NBT) substrate (B1911-100ML) was purchased from (Sigma-Aldrich Co, USA).

### 2.11. Statistical analysis

The graphical presentation of the data in this study was from biological replicates and presented as the mean ± SD done using ordinary one-way ANOVA with Tukey's multiple comparisons test by GraphPad Prism software (version 9.4.0, CA, USA). The significance was represented as ^*^p < 0.05 and ^**^p < 0.01, ^***^p < 0.001, ^****^p < 0.0001, and ns (non-statically significant).

## 3. Results

### 3.1. Structure description

The structures of sirtuins retrieved from RCSB were scrutinized carefully. Multiple deposits of protein structure were found for the six sirtuin isoforms. The PDB structures 4I5I(Sirt1), 4RMH (Sirt2), 4JSR(Sirt3), 6LJK(Sirt5), 3K35(Sirt6), and 5IQZ(Sirt7) were selected because of better resolution and with zero mutation and superimposed to analyze the similarity and efficiency against all the deposited structures using Pymol software (PyMOL Molecular Graphics System, Version 2.5.5, Schrödinger, LLC). The binding pockets and the interactive amino acids present in the pockets were also visualized. While preparing the sirtuin protein, essential Zn^+^ metal and the NAD^+^ bound to the protein were allowed to be present within the structure, and the ligand of interest was docked against the binding site.

### 3.2. Molecular docking

**Figure 2:**
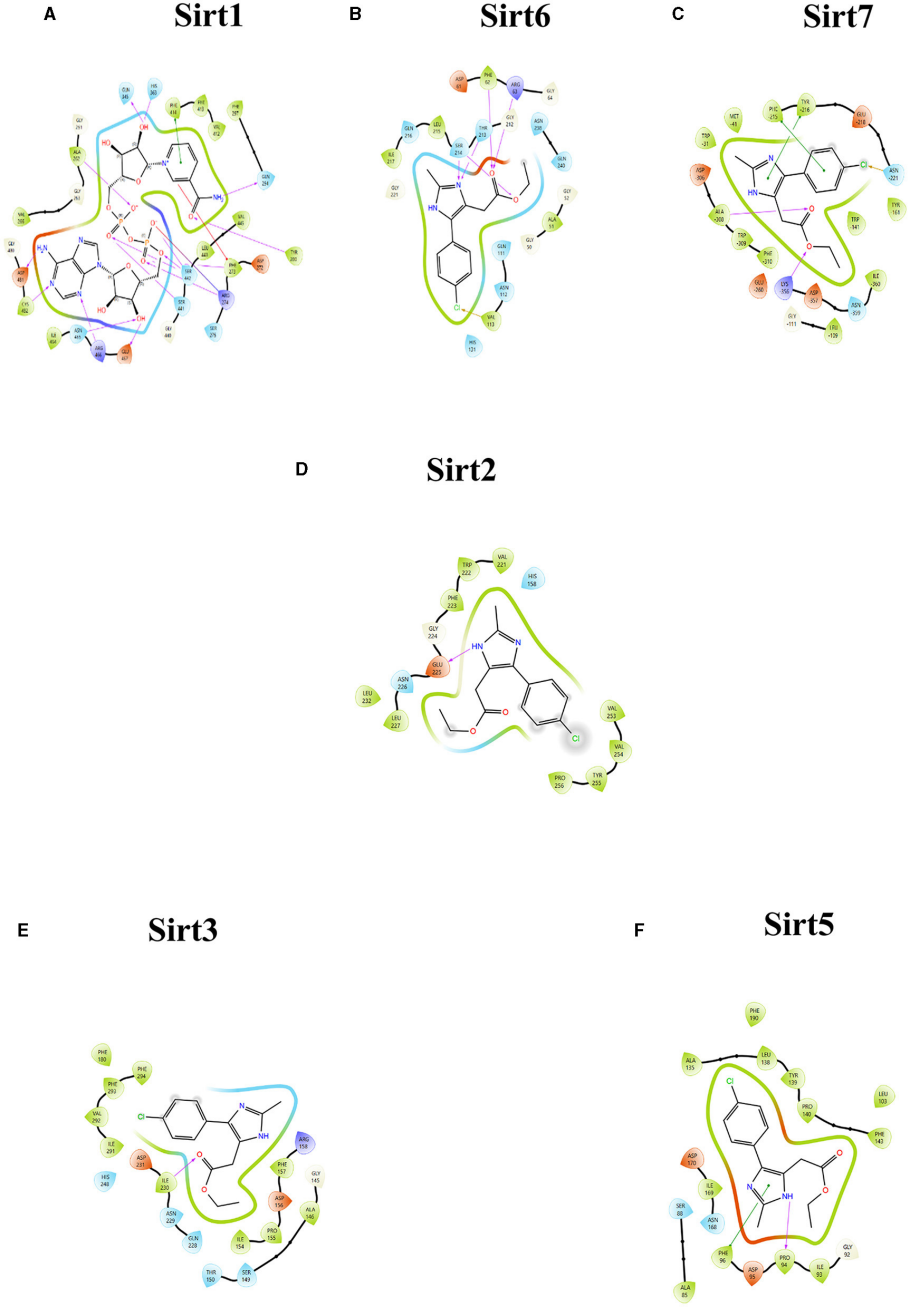
Ligand interaction between the sirtuins and the imidazole derivative. **(A–C)** Are the interactions of nuclear sirtuins Sirt1, Sirt6, and Sirt7, respectively. **(D)** Shows the ligand interaction with cytoplasmic sirtuin Sirt2. **(E, F)** Show the interaction of the mitochondrial sirtuins Sirt3 and Sirt5 with the ligand. The pink arrow indicates the hydrogen bonds and the green line depicts the pi-pi interaction formed between the ligand and the protein.

**Figure 3:**
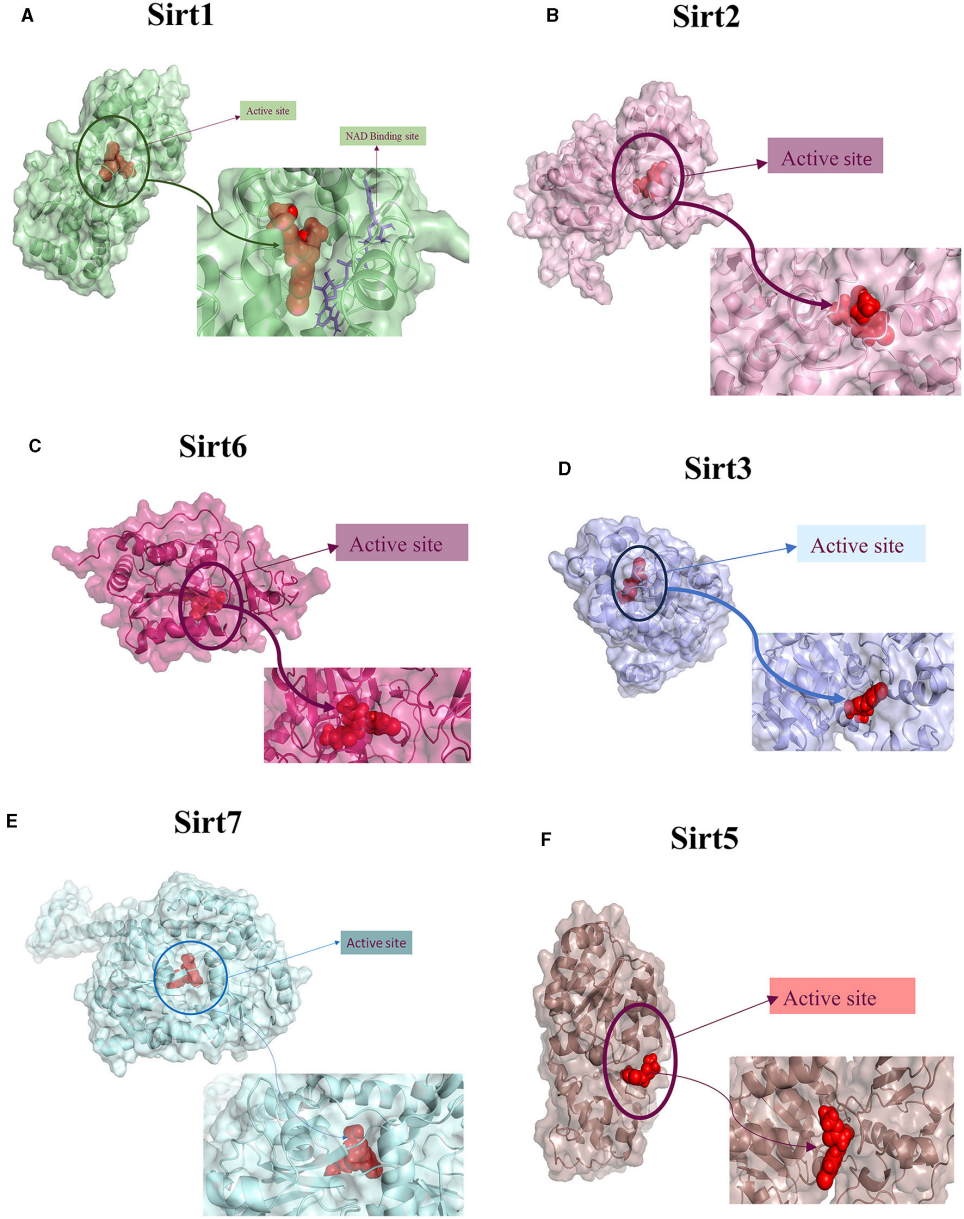
Protein-ligand interaction. 3D view of ligand fit into the binding pocket of the protein. The ligands bound to the active site of the protein are shown. **(A)** Sirt1, **(B)** Sirt2, **(C)** Sirt6, **(D)** Sirt3, **(E)** Sirt7, and **(F)** Sirt5.

A molecular docking experiment was carried out using the Glide software in which the selected protein PDB was docked against the selected imidazole derivatives. Glide incorporates various scoring functions to assess the binding affinities of ligands within the active site (Figures 2, 3). These scoring functions consider factors such as van der Waals interactions, hydrogen bonding, electrostatic interactions, and hydrophobic interactions. Among all the compounds subjected to the docking, Ethyl 2-[5-(4-chlorophenyl)-2-methyl-1-H-Imidazole-4-yl] acetate showed higher docking score, glide score, and glide energy. Furthermore, the compound also had impressive interactions with all the sirtuins, with some concern for cellular localization (Table 2).

**Table 2:** Molecular docking analysis—Glide Module.

**PDB**	**Sirt1 (4I5I)**		**Sirt2 (4RMH)**		**Sirt3 (4JSR)**		**Sirt5 (6LJK)**		**Sirt6 (3K35)**		**Sirt7 (5IQZ)**	
**Ligand Name**	**Glide Score; kcal/mol**	**Glide Energy; kcal/mol**	**Glide Score; kcal/mol**	**Glide Energy; kcal/mol**	**Glide Score; kcal/mol**	**Glide Energy; kcal/mol**	**Glide Score; kcal/mol**	**Glide Energy; kcal/mol**	**Glide Score; kcal/mol**	**Glide Energy; kcal/mol**	**Glide Score; kcal/mol**	**Glide Energy; kcal/mol**
Ethyl 2-(2,5-diphenyl-1H-imidazole-4-yl) acetate	−5.727	−18.208	−4.32	−14.778	−4.495	−17.511	−7.053	−15.008	−5.401	−19.483	−6.229	−41.320
Ethyl 2-[2-phenyl-5-(3,4,5-trimethoxyphenyl)-1H-imidazol-4-yl] acetate	−6.655	−28.841	−7.289	−39.668	−8.186	−54.470	−4.912	−35.961	−5.773	−47.432	−5.000	−41.870
Ethyl 2-[5-(4-chlorophenyl)-2-phenyl-1H-imidazol-4-yl] acetate	−7.556	−31.593	−9.056	−46.782	−7.019	−44.698	−5.735	−32.778	−5.225	−45.219	−5.498	−42.018
Ethyl 2-[5-(4-bromophenyl)-2-phenyl-1H-imidazol-4-yl] acetate	−7.742	−35.492	−8.816	−46.547	−7.442	−49.158	−4.686	−32.981	−4.664	−44.797	−5.404	−42.510
Ethyl 2-{2-phenyl-5-[4-(trifluoromethyl) phenyl]-1H-imidazol-4-yl} acetate	−9.656	−26.563	−7.711	−39.699	−7.714	−49.695	−5.584	−32.798	−4.825	−45.74	−5.558	−44.785
Ethyl 2-[5-(4-chlorophenyl)-2-methyl-1-H-Imidazole-4-yl) acetate	−7.807	−37.735	−8.835	−41.366	−7.053	−43.362	−5.053	−31.857	−6.544	−45.913	−5.616	−39.446
Imidazole	−5.723	−18.112	−4.318	−14.734	−4.495	−17.511	−5.277	−15.008	−5.385	−19.497	−4.092	−16.893

### 3.3. Molecular dynamics

**Figure 4:**
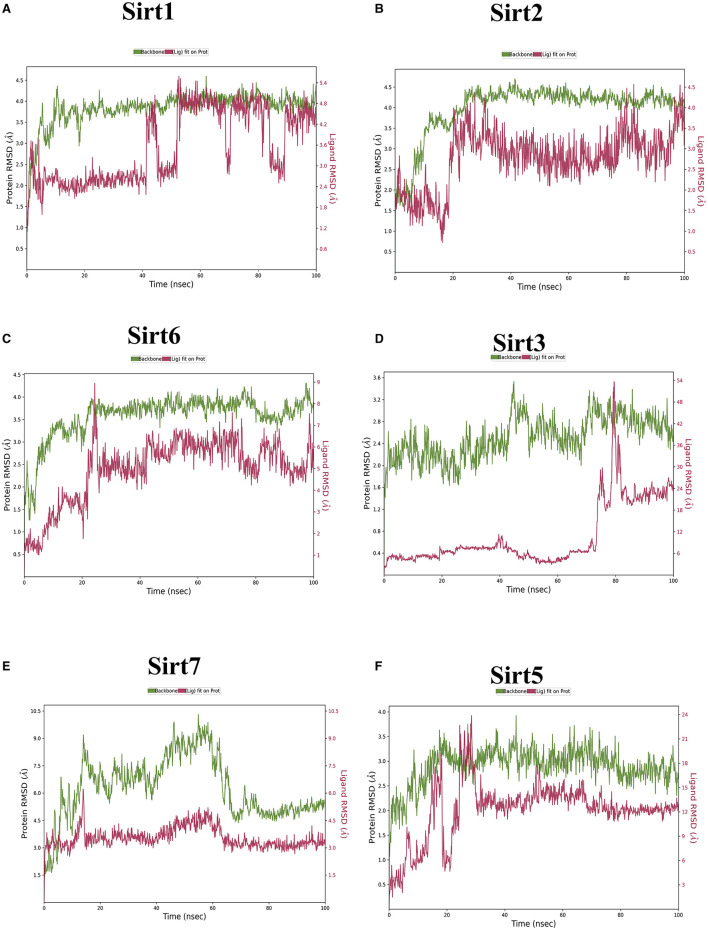
The root mean square deviation (RMSD). RMSD is a metric used to gauge the average change in atom displacement within a selected group of atoms in a specific frame in comparison to a reference frame. This calculation was performed for all frames in a given trajectory. The plot illustrates the progression of RMSD for the sirtuin protein (displayed on the left Y-axis in each graph plot). Meanwhile, ligand RMSD (depicted on the right Y-axis) provides insight into the stability of the ligand concerning the protein and its binding pocket. **(A)** Sirt1-ligand RMSD; **(B)** Sirt2-ligand RMSD; **(C)** Sirt6-ligand RMSD; **(D)** Sirt3-ligand RMSD; **(E)** Sirt7-ligand RMSD; and **(F)** Sirt5-ligand RMSD.

**Figure 5:**
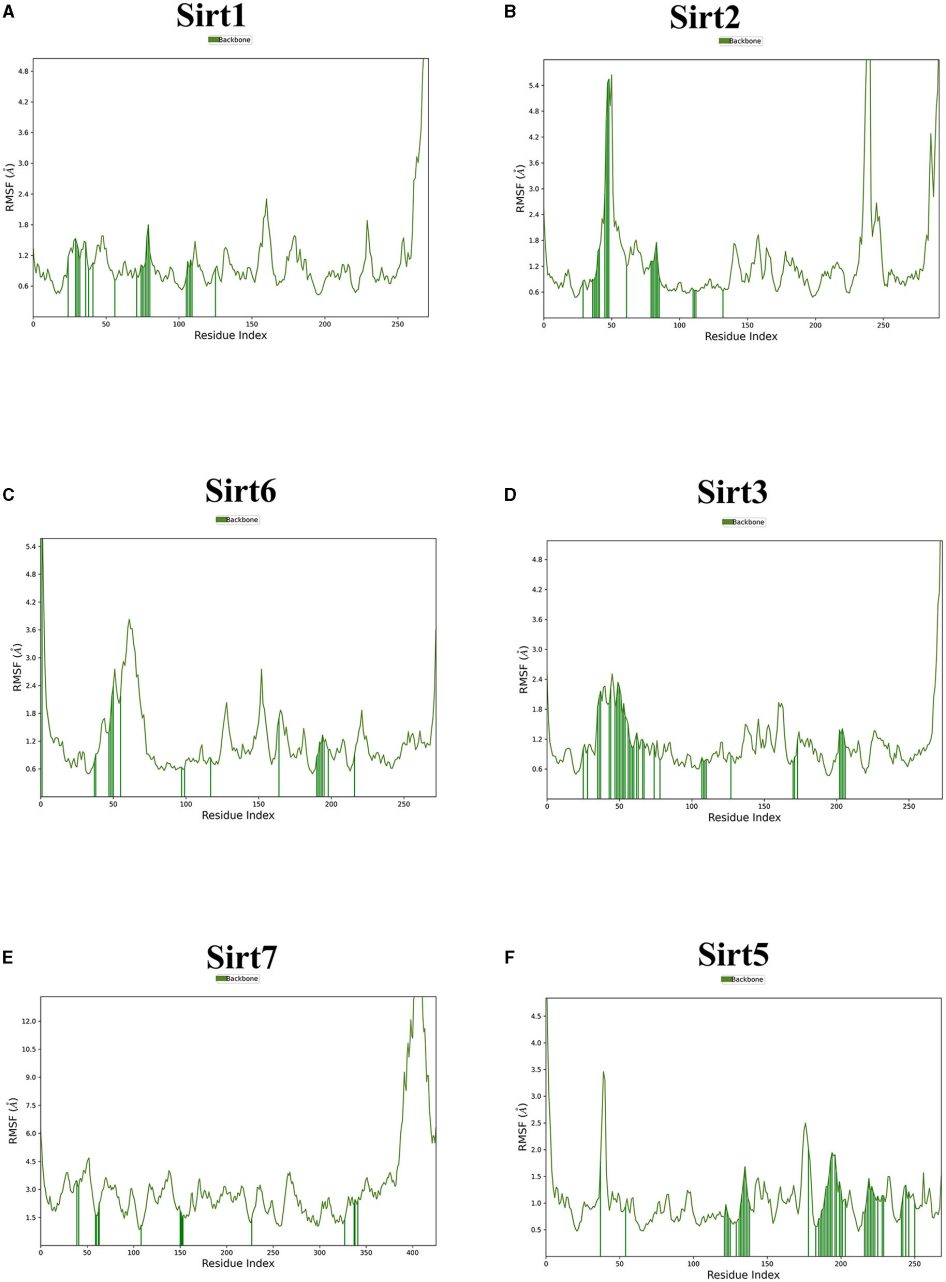
The root mean square fluctuation (RMSF). The parameter is useful for characterizing local changes along the protein chain. Sirtuin protein residues that interact with the ligand are marked with green-colored vertical bars. **(A)** Sirt1-ligand RMSF; **(B)** Sirt2-ligand RMSF; **(C)** Sirt6-ligand RMSF; **(D)** Sirt3-ligand RMSF; **(E)** Sirt7-ligand RMSF; and **(F)** Sirt5 -ligand RMSF.

**Figure 6:**
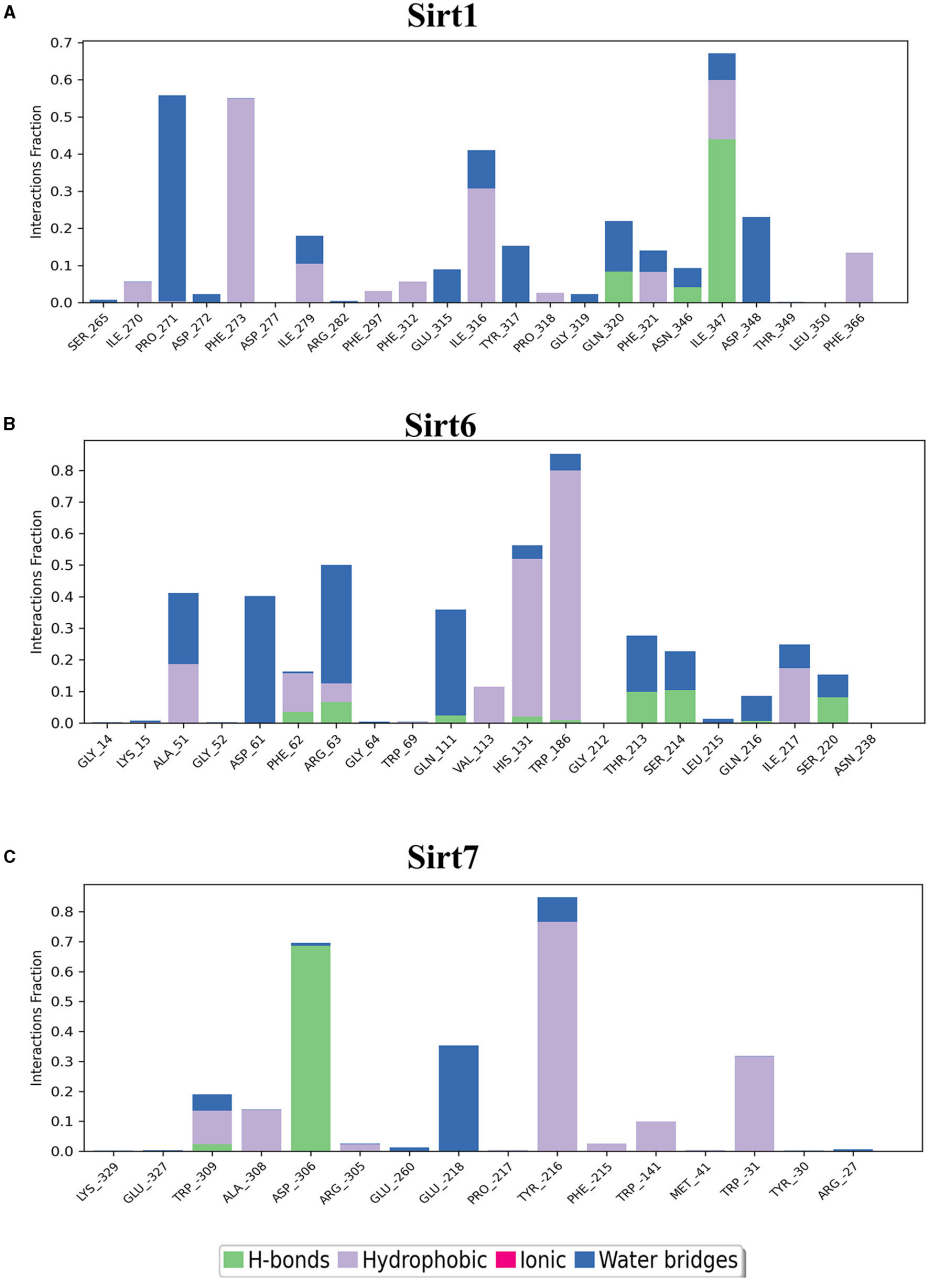
Nuclear sirtuin-ligand interaction. Protein interactions with the ligand can be monitored throughout simulations. These interactions can be categorized by type and summarized, as shown in the plot, which depicts hydrogen bonds and hydrophobic, ionic, and water bridges. **(A)** Sirt1, **(B)** Sirt6, and **(C)** Sirt7.

**Figure 7:**
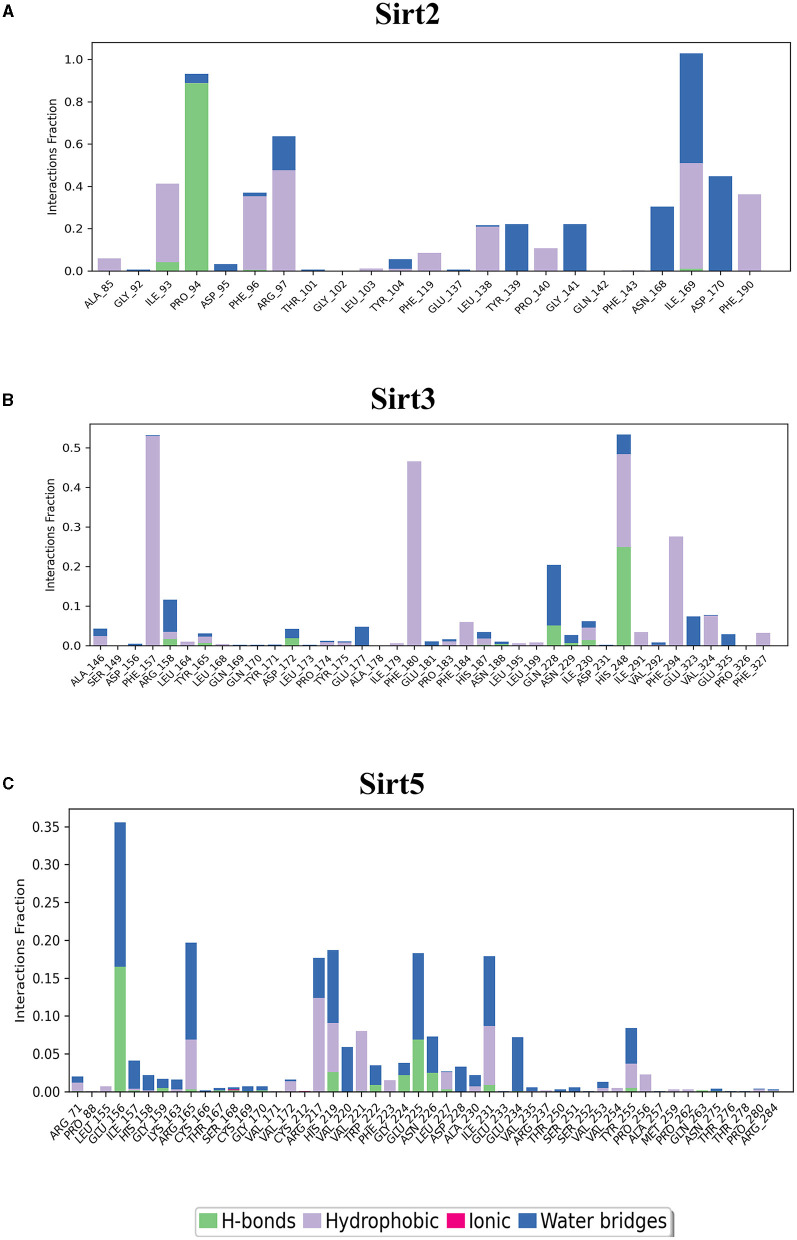
Cytoplasmic and mitochondrial sirtuin-ligand interaction. Protein interactions with the ligand can be monitored throughout simulations. These interactions can be categorized by type and summarized, as shown in the plot, which depicts hydrogen bonds and hydrophobic, ionic, and water bridges. **(A)** Sirt2; **(B)** Sirt3; **(C)** Sirt5.

MDS run with 100 ns simulation and 1000 frames generated a set of RMSD (Figure 4), RMSF (Figure 5), and protein-ligand interaction plots (Figures 6, 7). Sirt1, Sirt2, and Sirt6 were found to have stable simulation ranges with the increase of nanoseconds. Other sirtuins showed non-synchronous simulation stability.

### 3.4. DFT calculation

**Figure 8:**
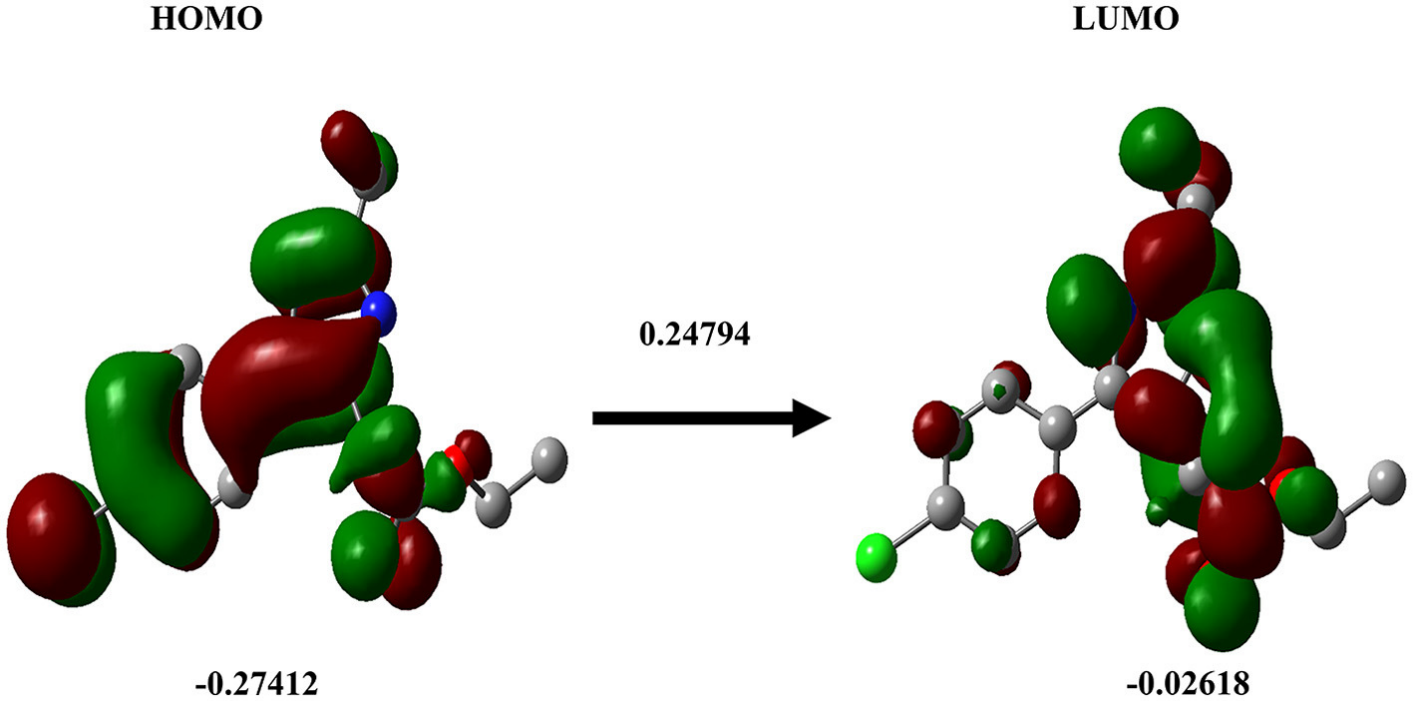
Density functional theory. The DFT calculation for the ligand Ethyl 2-[5-(4-chlorophenyl)-2-methyl-1-H-Imidazole-4-yl) acetate is shown. HOMO **(left)**, LUMO **(right)**.

DFT analysis is a promising method to investigate protein-ligand interactions from an atomistic-electronic perspective. It was carried out to understand the structure and relative energy of the ligand in the binding pocket as well as the electronic rearrangements that occur upon ligand binding. We also calculated the highest occupied molecular orbital of the ligand, which was approximately−0.27412, and the least unoccupied molecular orbital was−0.02618 (Figure 8).

### 3.5. ADME analysis

ADME properties were analyzed for all the ligands that were investigated in this study. The results of the ADME analysis showed effective absorption, solubility, and the ability of the compound to dwell in the gut-blood barrier and blood-brain barrier (Table 3).

**Table 3:** ADMET analysis of the imidazole derivatives in comparison with imidazole parent structure emphasizing the efficiency of imidazole derivatives.

**Ligand name**	**Molecular weight**	**SASA**	**FOSA**	**FISA**	**Donor HB**	**Acceptor HB**	**VOLUME**	**QPlogPw**	**QPlogP/ w**	**QPlogBB**	**QPlogS**	**QPPCaCO**	**Human oral absorption**	**%Human oral absorption**	**Rule of Five**
Acceptable Range	(< 500 Da)	(300.0 −1000.0)	(0.0 – 750.0)	(7.0 – 330.0)	(< 5), g)	(< 10), h)	(500.0 – 2000.0)	(4.0 – 45.0)	(−2.0-6.5)	(−3.0 – 1.2)	(−6.5-0.5)	(< 25 poor, >500 great)	—-	(< 25% is poor, >80% is high)	maximum is 4
Ethyl 2-(2,5-diphenyl-1H-imidazole-4-yl) acetate	306.363	585.302	143.504	73.186	1	3.5	1029.252	8.373	4.212	−5.184	−0.284	2102.639	3	100	0
Ethyl 2-[2-phenyl-5-(3,4,5-trimethoxyphenyl)-1H-imidazol-4-yl] acetate	396.442	683.134	374.559	72.031	0	4.75	1255.511	6.817	4.951	−5.699	−0.506	2055.082	3	100	0
Ethyl 2-[5-(4-chlorophenyl)-2-phenyl-1H-imidazol-4-yl] acetate	340.808	646.409	176.469	57.976	1	3.5	1111.022	8.259	5.05	−6.598	−0.041	2793.266	1	100	1
Ethyl 2-[5-(4-bromophenyl)-2-phenyl-1H-imidazol-4-yl] acetate	385.259	654.421	177.218	57.77	1	3.5	1121.383	8.291	5.14	−6.768	−0.033	2805.877	1	100	1
Ethyl 2-{2-phenyl-5-[4-(trifluoromethyl) phenyl]-1H-imidazol-4-yl} acetate	374.362	666.352	171.63	59.5	1	3.5	1160.555	8.18	5.504	−7.159	0.054	2701.827	1	100	1
Ethyl 2-[5-(4-chlorophenyl)-2-methyl-1-H-Imidazole-4-yl) acetate	278.738	558.092	272.468	72.352	1	3.5	933.926	6.793	3.6	−5.01	−0.145	2040.747	3	100	0
Imidazole	68.078	228.199	0	65.325	1	2	309.474	5.255	−0.08	−0.485	0.1	2379.132	3	86.91	0

### 3.6. Cytotoxicity effect of Ethyl 2-[5-(4-chlorophenyl)-2-methyl-1-h-imidazole-4-yl] acetate

**Figure 9:**
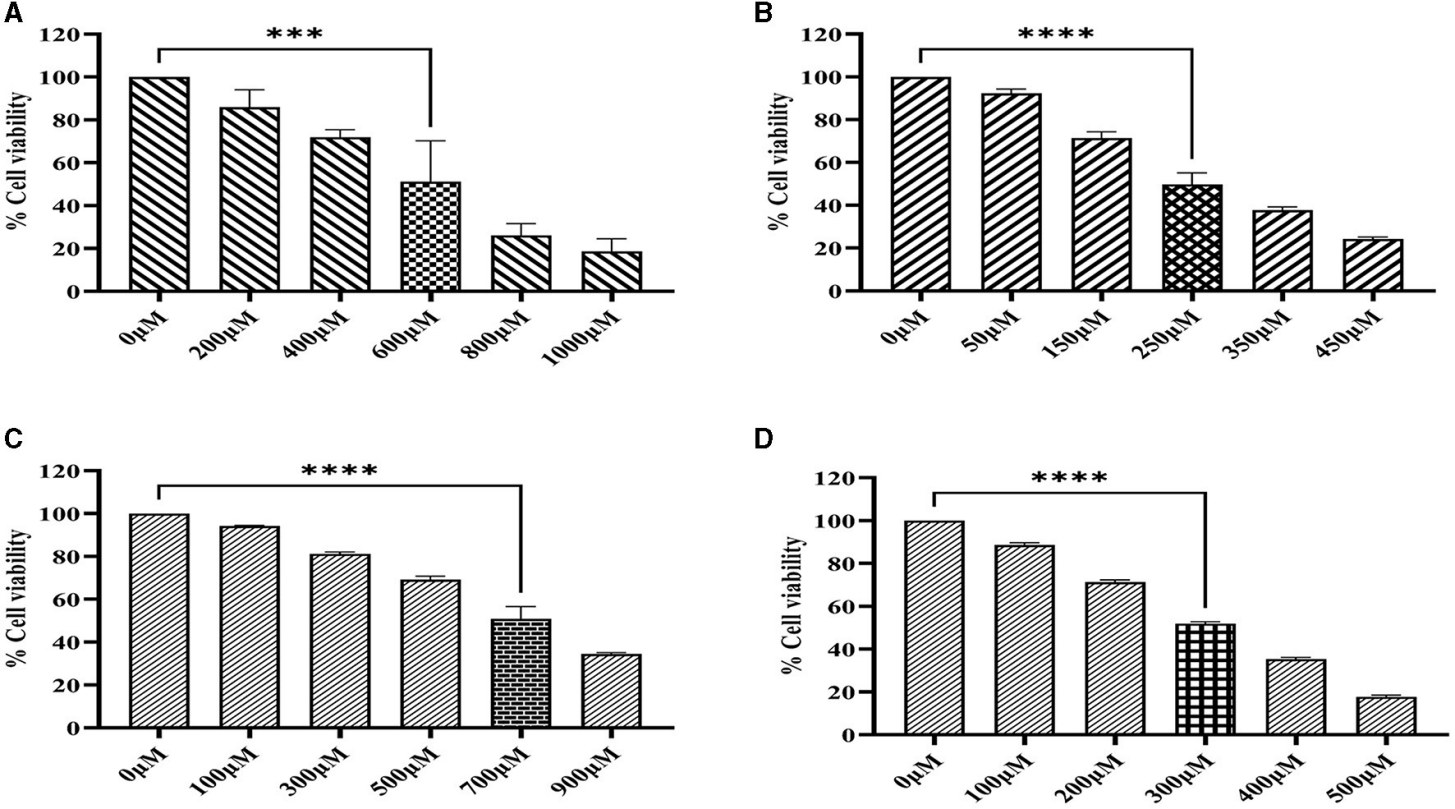
MTT assay. The cell viability activity of imidazole and imidazole derivative Ethyl 2-[5-(4-chlorophenyl)-2-methyl-1-H-Imidazole-4-yl) acetate with different concertation for a treatment period of 24 h in NSCLC cell lines A549 and NCI-H460 cell lines. **(A, B)** Shows the cell viability activity of imidazole and imidazole derivatives in the A549 cell line. Whereas **(C, D)** show the cell viability activity of imidazole and imidazole derivatives in the NCI-H460 cell line. Data represent mean values ± SD. ***p < 0.001 and ****p < 0.0001.

The cytotoxicity of imidazole and its derivative Ethyl 2-[5-(4-chlorophenyl)-2-methyl-1-H-Imidazole-4-yl] acetate was determined in NSCLC cell lines A549 and NCI-H460 using MTT assay. The cells were treated with imidazole and imidazole derivatives of different concentrations for 24 h. The cell viability decreased gradually as concentration increased. The half maximal inhibitory concentration (IC_50_) of imidazole and its derivative was found to be 600 μM and 250 μM in the A549 cell line (Figures 9A, B) and 700 μM and 300 μM in the NCI-H460 cell line (Figures 9C, D). The imidazole derivative at lower concentrations reduced the cell viability compared to the parent compound imidazole in the NSCLC cell lines.

### 3.7. *In-vitro* analysis of the imidazole derivative Ethyl 2-[5-(4-chlorophenyl)-2-methyl-1-h-imidazole-4-yl] acetate on class III histone deacetylase family (Sirt)

**Figure 10:**
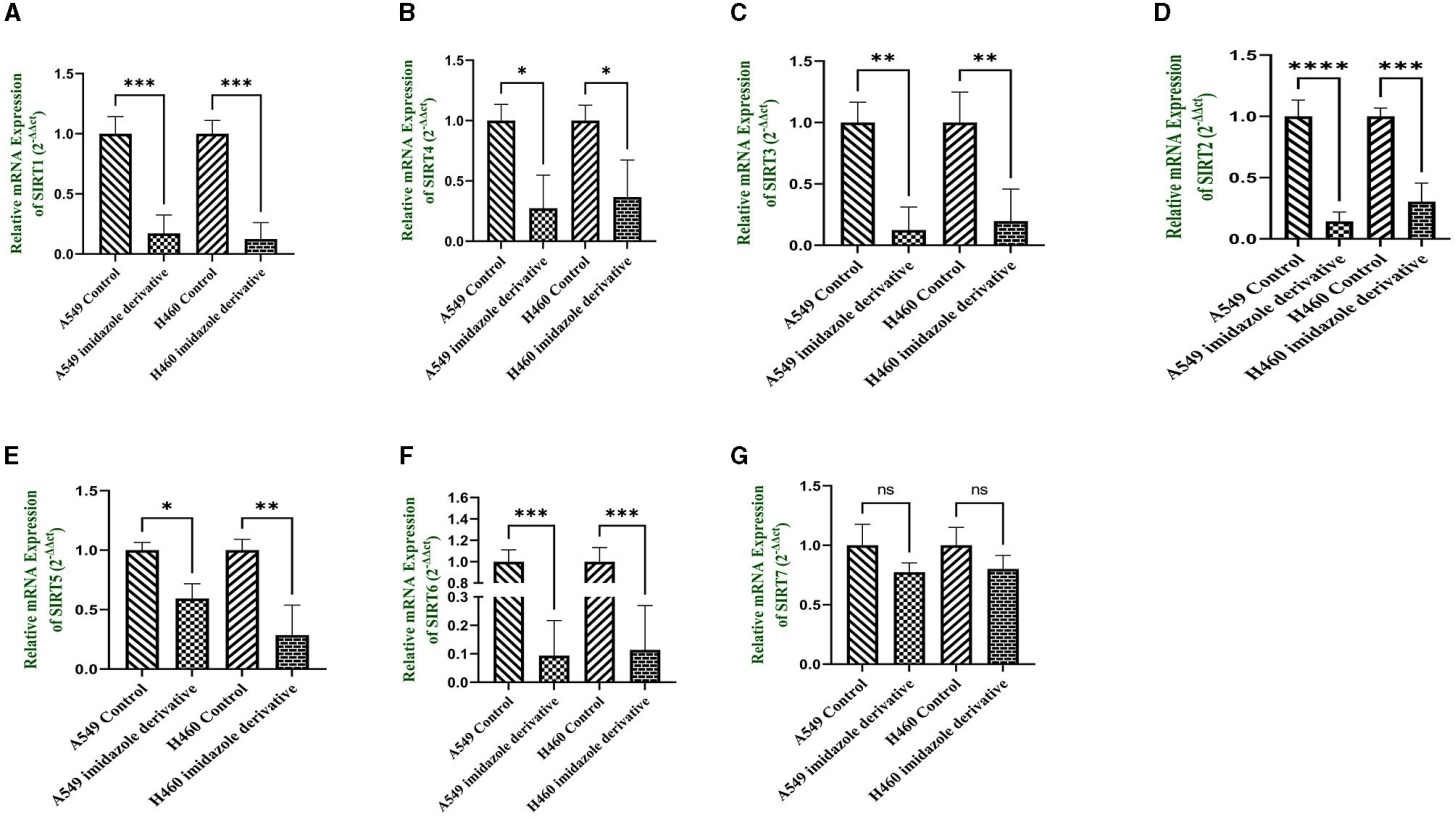
mRNA expression. Gene expression of the Class III histone deacetylase family (Sirt 1-7) was performed in quantitative Real-time PCR. The graphical representation shows expression of different sirtuins **(A)** Sirt1, **(B)** Sirt2, **(C)** Sirt3, **(D)** Sirt4, **(E)** Sirt5, **(F)** Sirt6, and **(G)** Sirt7 in NSCLC cell lines A549 and NCI-H460 (Data represents mean values ± SD. *p < 0.05, **p < 0.01, ***p < 0.001, ****p < 0.0001, and ns, non-statistically significant).

**Figure 11:**
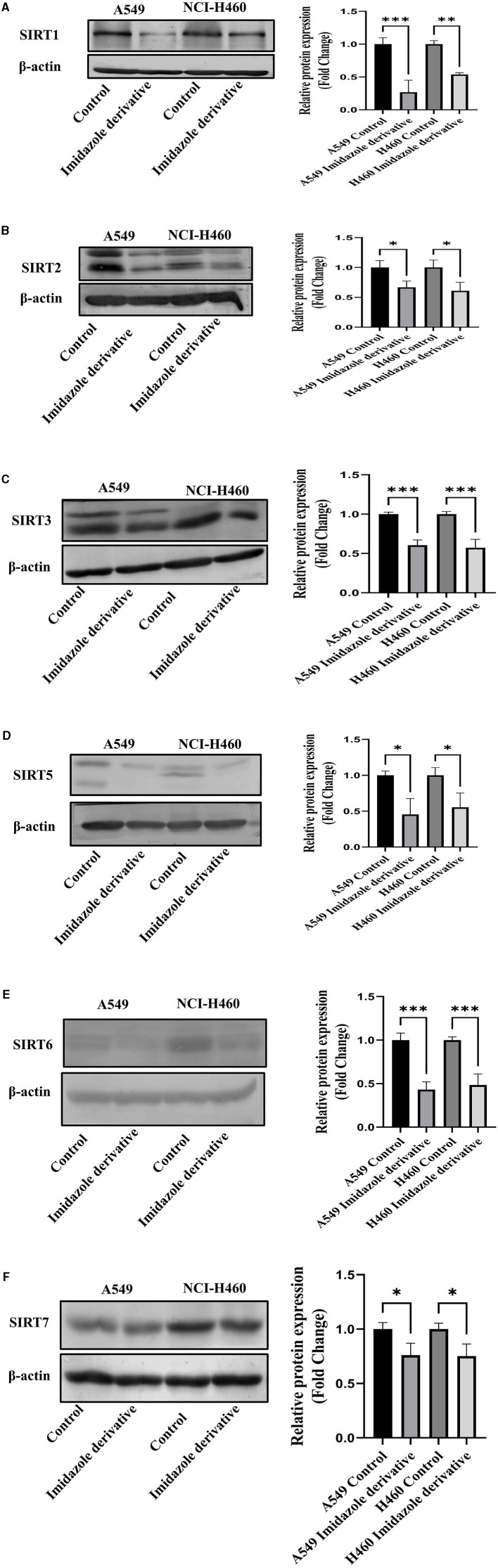
Protein expression. Western blotting was performed to understand the effect of Ethyl 2-[5-(4-chlorophenyl)-2-methyl-1-H-Imidazole-4-yl) acetate on protein expression of sirtuins. The results show the decrease in expression of **(A)** Sirt1, **(B)** Sirt2, **(C)** Sirt3, **(D)** Sirt5, **(E)** Sirt6, and **(F)** Sirt7 in Ethyl 2-[5-(4-chlorophenyl)-2-methyl-1-H-Imidazole-4-yl) acetate treated NSCLC cell lines A549 and NCI-H460 in comparison with control cells (untreated). β-actin was used as an endogenous control for normalization. Data represents mean values ± SD. *p < 0.05, **p < 0.01, and ***p < 0.001.

Sirtuins are class III histone deacetylases with seven isoforms (Sirt 1-7). The role of sirtuins varies differently depending on the type of cancer. In this study, we explored the effect of imidazole derivative Ethyl 2-[5-(4-chlorophenSirtSirtyl)-2-methyl-1-H-Imidazole-4-yl] acetate on NSCLC cell line A549 and NCI-H460 cell lines. The gene and protein expression studies were carried out to understand the effect of the imidazole derivative. The results from the gene expression studies confirmed the downregulation of sirtuins on treatment with Ethyl 2-[5-(4-chlorophenyl)-2-methyl-1-H-Imidazole-4-yl] acetate. Among all the sirtuins, Sirt1, Sirt2, and Sirt6 showed a prominent decrease in expression, followed by Sirt3, Sirt5, and Sirt4 in that order. Sirt7 was decreased non-significantly (Figure 10). Further Western blotting analysis was carried out to confirm the effect of Ethyl 2-[5-(4-chlorophenyl)-2-methyl-1-H-Imidazole-4-yl] acetate at the protein level. The expression of all the sirtuin members was decreased on treatment with Ethyl 2-[5-(4-chlorophenyl)-2-methyl-1-H-Imidazole-4-yl] acetate. Among the different isoforms of sirtuins, Sirt1 and Sirt6 were greatly affected, followed by Sirt2, Sirt5, Sirt3, and Sirt7. Figure 11 shows the Western blot of all the isoforms of sirtuins. The decreased expression of Sirt1 was different in A549 and H460 cell lines, whereas Sirt6 was decreased to the same extent in A549 and NCI-H460 cell lines.

## 4. Discussion

The imidazole derivatives, designed and synthesized in-house, were subjected to Glide docking analysis. We found that in spite of the cellular localization of the sirtuins, all the compound structures interacted with the protein, especially compound Ethyl 2-[5-(4-chlorophenyl)-2-methyl-1-H-Imidazole-4-yl] acetate, which assured binding with all six sirtuin proteins. The glide gscore indicated that this compound had more affinity toward nuclear sirtuins Sirt1, Sirt6, and Sirt7 (**−7.807**, **−6.544, and** **−5.616**) and cytosolic sirtuin Sirt2 (**−8.835**).

The binding pockets of the protein comprised a set of amino acids (isoleucine, alanine, phenylalanine, tyrosine, tryptophan, glutamate, histidine, arginine, and aspartate) that form effective hydrogen bonds and pi-pi interaction with the ligand. Sirt1, Sirt6, Sirt7, and Sirt2 showed more hydrogen bond interactions, whereas the mitochondrial sirtuins Sirt3 and Sirt5 possessed the least interactive bonds.

Using the RMSD data, we determined the average distance between atoms in a simulated protein-ligand structure and a reference protein structure (23). This quantified the structural changes in the molecule during the simulation, commonly used to assess simulation stability and convergence. The RMSF values were also calculated to understand the average deviation of atom positions from their mean during the simulation, providing insights into the flexibility and dynamic behavior of various biomolecule regions (24).

The MD simulations investigated ligand interactions and, in particular, examined how a small molecule (ligand) interacted with a larger biomolecule (e.g., protein or nucleic acid). These ligand interactions encompassed hydrogen bonding, van der Waals forces, and electrostatic interactions, and understanding these interactions offered insights into binding strength, binding sites, and potential mechanisms of action. Using trajectory analysis, the complex RMSD for nuclear, mitochondrial, and cytoplasmic sirtuins was determined. The RMSD range for the sirtuin protein-ligand Ethyl 2-[5-(4-chlorophenyl)-2-methyl-1-H-Imidazole-4-yl) acetate complex was within 4.0 Å. All the RMSD graphs indicated that our compound had attained equilibrium and stayed at approximately 4 Å throughout the 100 ns simulation. This demonstrated the ability of our compound to effectively be stable-bound with all six sirtuins employed in the study.

Sirtuins are interconnected HDAC enzymes, and they take turns to achieve pathway activation and inhibition. For instance, multiple studies have reported that Sirt1 possesses power over other nuclear sirtuins. In the case of double-stranded break repair mechanisms, Sirt1 facilitates the mobilization of Sirt6 to double-strand breaks by deacetylation (25). One report suggested that Sirt1 and Sirt6 can bind to the N and C terminals of the Suv39h protein and regulate the repressor pathway of IkBα expression (26). In contrast, there are research reports that emphasize the importance of Sirt6 in regulating the random acetylation of Sirt1 with respect to lung cancer. Sirt1 can acetylate or deacetylate and regulate multiple pathways in lung cancer, like acetylation of AMP (27), AKT (28), PPAR γ (29), and more. Our gene expression and protein expression data suggest that the compound Ethyl 2-[5-(4-chlorophenyl)-2-methyl-1-H-Imidazole-4-yl] acetate cooperated well with nuclear sirtuins, which could favor the inhibition of nuclear sirtuins and activation of tumor suppression pathways. Inhibition of nuclear sirtuins can clearly influence the actions of other sirtuins.

The HOMO energy level represents the energy of the highest electron orbital that contains electrons in the molecule. It signifies the energy required to remove an electron from the highest occupied orbital. A lower (more negative) HOMO energy indicates that electrons are relatively tightly bound, which implies that the molecule is less likely to readily donate electrons. In terms of chemical reactivity, the HOMO energy level is associated with the molecule's ability to act as an electron donor in chemical reactions (30, 31). In our study, all the selected imidazole derivatives demonstrated values that surpassed the defined range. Based on a comprehensive analysis, it can be concluded that the compounds showed promising potential in terms of permeability. The ADME analysis revealed that all the compounds investigated in the study possessed favorable drug-likeness and did not violate Lipinski's rule of five. As a result, all the compounds were included in the docking study. From the ADME analysis and the docking score, it is evident that the imidazole derivative Ethyl 2-[5-(4-chlorophenyl)-2-methyl-1-H-Imidazole-4-yl] acetate has greater interaction with all six sirtuins compared to imidazole and its other derivatives.

In the surface area analysis, several crucial components were assessed, including the total solvent accessible surface area (SASA), which accounts for the overall surface area available to solvents. Additionally, the hydrophobic component of SASA, known as FOSA, and its counterpart, the hydrophilic component called FISA, were examined (32). The evaluation of these components yielded values that largely fell within an acceptable range, as stipulated in the Qikprop manual provided by Schrödinger. In particular, the software's guidelines were referenced to ensure that the values obtained for SASA, FOSA, FISA, PISA, and volume align with established standards. Furthermore, the predictive permeability of the compounds was a focal point of investigation. This was achieved through the utilization of two key metrics: QPPCaco, a model designed for assessing gut-blood barrier permeability (33), and QPlogBB, which pertains to the brain-blood partition coefficient (34). QPPCaco values exceeding 500 are indicative of favorable permeability characteristics. Our investigation revealed that all the selected imidazole derivatives had values that surpassed the defined range. Based on a comprehensive analysis. It can be concluded that the compounds showed promising potential in terms of permeability. The ADME analysis and the docking score established that the imidazole derivative, Ethyl 2-[5-(4-chlorophenyl)-2-methyl-1-H-Imidazole-4-yl] acetate, had greater interaction with the sirtuins family compared to the imidazole.

Based on the Insilco result, we carried out *in-vitro* studies with Ethyl 2-[5-(4-chlorophenyl)-2-methyl-1-H-Imidazole-4-yl] acetate to explore its inhibitory effect on sirtuin family members (Sirt 1-7). Initially, we undertook an MTT assay to fix the IC_50_ concentration of the Ethyl 2-[5-(4-chlorophenyl)-2-methyl-1-H-Imidazole-4-yl] acetate. An analysis of the respective MTT results for imidazole and imidazole derivative revealed that the imidazole derivative had a higher effect at lower concentrations in 24 h of treatment in A549 (250 μM) and NCI-H460 (300 μM) compared to the parent imidazole compound in A549 (600 μM) and NCI-H460 (700 μM) cell lines. Furthermore, gene and protein expression studies were carried out with IC_50_ concentration of imidazole derivative Ethyl 2-[5-(4-chlorophenyl)-2-methyl-1-H-Imidazole-4-yl] acetate. The gene expression results from qRT-PCR confirmed the decrease in the expression of sirtuins in NSCLC cell line A549 and NCI-H460.

Among the sirtuins, Sirt1, Sirt2, and Sirt6 were greatly reduced on treatment with Ethyl 2-[5-(4-chlorophenyl)-2-methyl-1-H-Imidazole-4-yl] acetate, comparted to Sirt3, Sirt5 and Sirt7. The results from the Western blot analysis also confirm the decreased expression of sirtuins, which correlated with the gene expression of sirtuins. Among the sirtuins, the protein expression of Sirt1 and Sirt6 was greatly reduced, which correlated with the gene expression of Sirt1 and Sirt6. A previous report from our lab crew explains that Sirt6 is highly expressed in NSCLC cell lines, and upon silencing Sirt6, cell cycle arrest and apoptosis are favored (35). Consequently, based on our current study, we believe that Ethyl 2-[5-(4-chlorophenyl)-2-methyl-1-H-Imidazole-4-yl] acetate potentially inhibits nuclear sirtuins, especially the expression of Sirt6 and regulates cancer progression.

## 5. Conclusion

Cancer is an indestructible target for biologists; every day, new drugs are out on the market. However, regulating compounds with potential are scarcely found. Our in-house compounds established potential interaction with epigenetic-modulating sirtuin enzymes that can inhibit cancer progression. Our *in-silico* data shows that the imidazole derivative Ethyl 2-[5-(4-chlorophenyl)-2-methyl-1-H-Imidazole-4-yl] acetate perfects the Lipinski rule of five and possesses a high docking score, which confirms the interaction with all sirtuin isoforms. The molecular dynamics data further confirms the stability of the bound ligand with the protein. Similar to the *in-silico* study, the results from the *in-vitro* study confirm the inhibitory effect of imidazole derivative Ethyl 2-[5-(4-chlorophenyl)-2-methyl-1-H-Imidazole-4-yl] acetate. Our study shows that the imidazole derivative is highly effective on nuclear sirtuins Sirt1 and Sirt6, as well as cytosolic sirtuin Sirt2. Furthermore, it has been found to be effective on other members of the sirtuins, namely Sirt3, Sirt5, and Sirt7, in both *in-silico* and *in-vitro* analysis. Therefore, based on the *in-silico* and *in-vitro* results, we claim that our in-house imidazole derivative Ethyl 2-[5-(4-chlorophenyl)-2-methyl-1-H-Imidazole-4-yl] acetate is a potential inhibitor of Class III HDACs, with particular reference to Sirt1, Sirt6, and Sirt2.

## Data availability statement

The original contributions presented in the study are included in the article/Supplementary material, further inquiries can be directed to the corresponding author/s.

## Ethics statement

Ethical approval was not required for the studies on humans in accordance with the local legislation and institutional requirements because only commercially available established cell lines were used. Ethical approval was not required for the studies on animals in accordance with the local legislation and institutional requirements because only commercially available established cell lines were used.

## Author contributions

UMRD: Conceptualization, Data curation, Formal analysis, Investigation, Methodology, Supervision, Validation, Writing—original draft, Writing—review and editing. SS: Methodology, Software, Validation, Writing—original draft. SA-G: Formal Analysis, Writing—review and editing. NA: Formal analysis, Writing—review and editing. SD: Formal analysis, Writing—review and editing. AA: Formal analysis, Writing—review and editing. MS: Formal analysis, Writing—review and editing. TR: Funding acquisition, Writing—review and editing. RV: Conceptualization, Funding acquisition, Project administration, Resources, Writing—original draft, Writing—review and editing.
