# Supplementary material for: In-silico and in-vitro functional validation of imidazole derivatives as potential sirtuin inhibitor
Source: Front Med (Lausanne). 2023 Nov 7;10:1282820. doi: 10.3389/fmed.2023.1282820 (PMC10662127; doi:10.3389/fmed.2023.1282820)

**Supplementary Data:**

Protein MDS allows us to observe the natural fluctuations, motions, and conformational changes that proteins undergo in their native state. Protein dynamics can influence ligand binding, so we performed MDS with only the PDB structure of the protein prepared using preparation wizard, Maestro version 13.2.128, Release 2022-2 and the system was built for the native protein and subjected to 100ns MD simulation using Desmond v7.0 with a relaxation time of 1ps and maintaining a consistent temperature of 300 K. Throughout the simulation, a series of 1000 frames were generated using a time step of 20ps.

**Supplementary
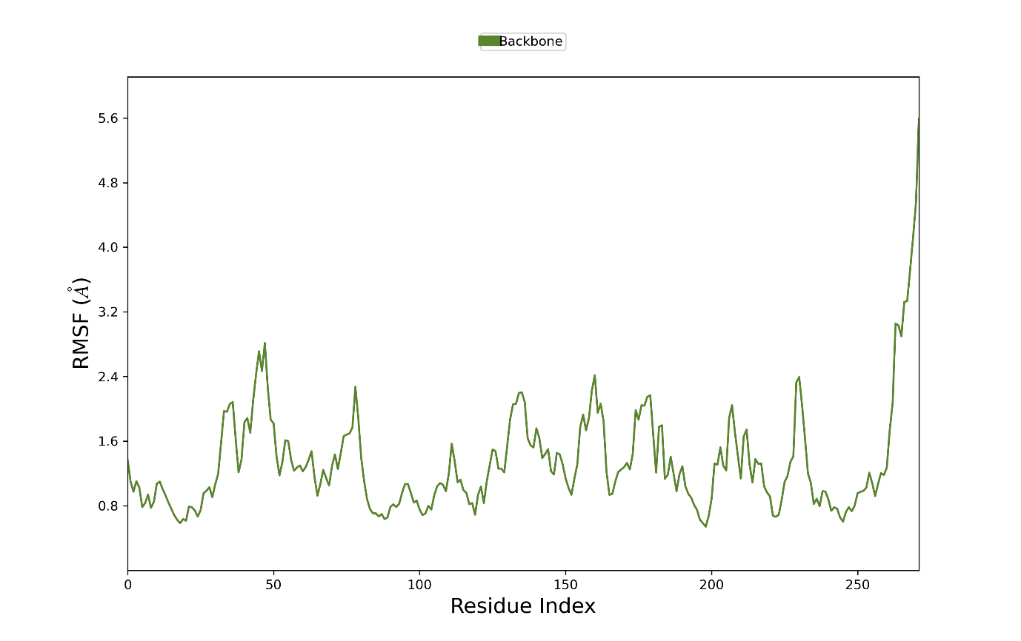

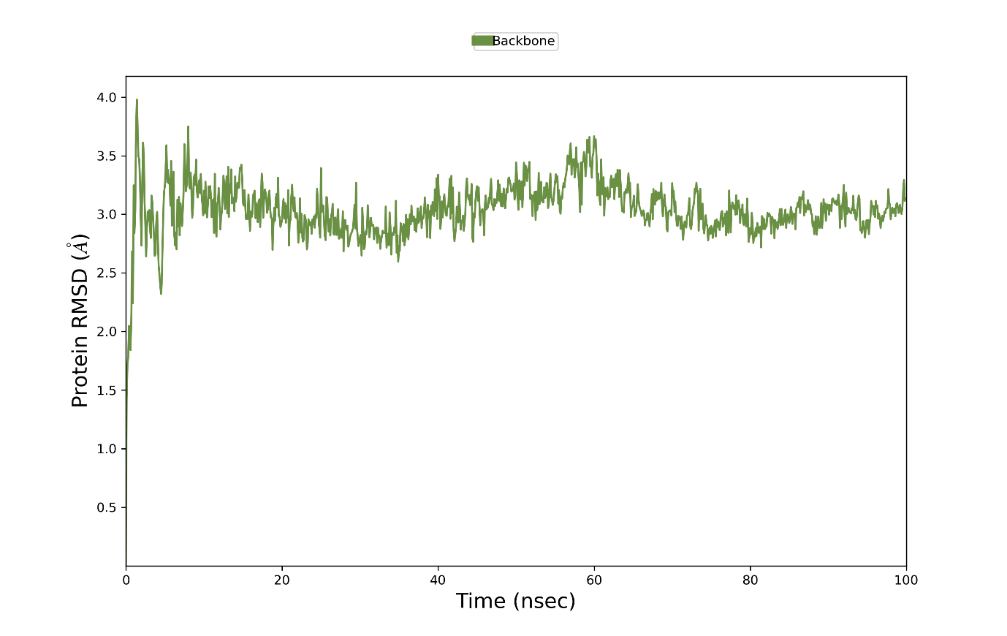
 Fig 1: Molecular Simulation of SIRT1 Protein**

**Supplementary
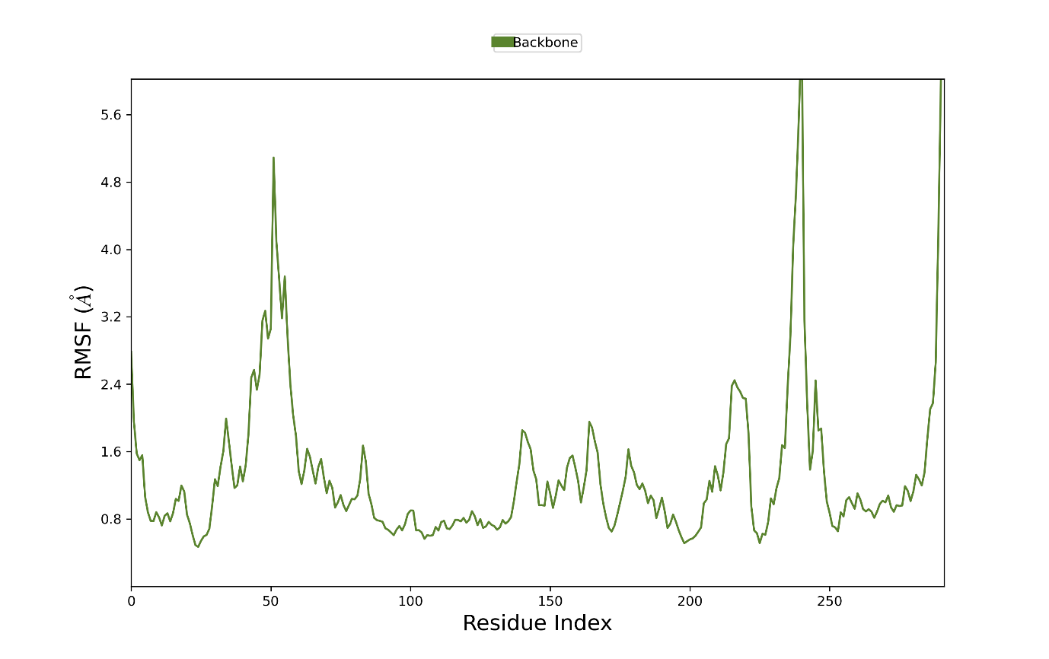

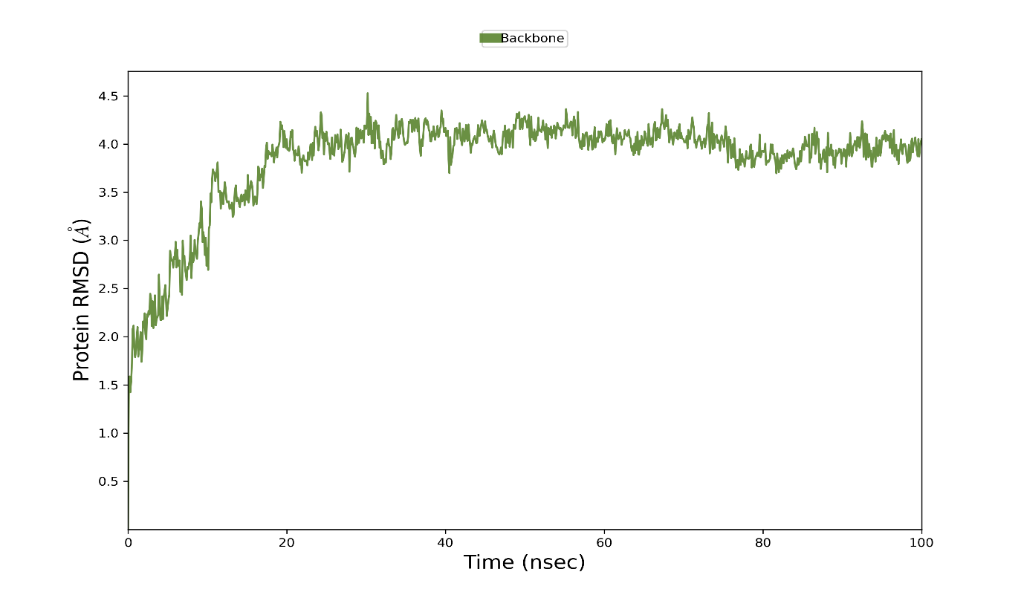
 Fig 2: Molecular Simulation of SIRT2 Protein**

**Supplementary Fig 3: Molecular Simulation of SIRT3 Protein**


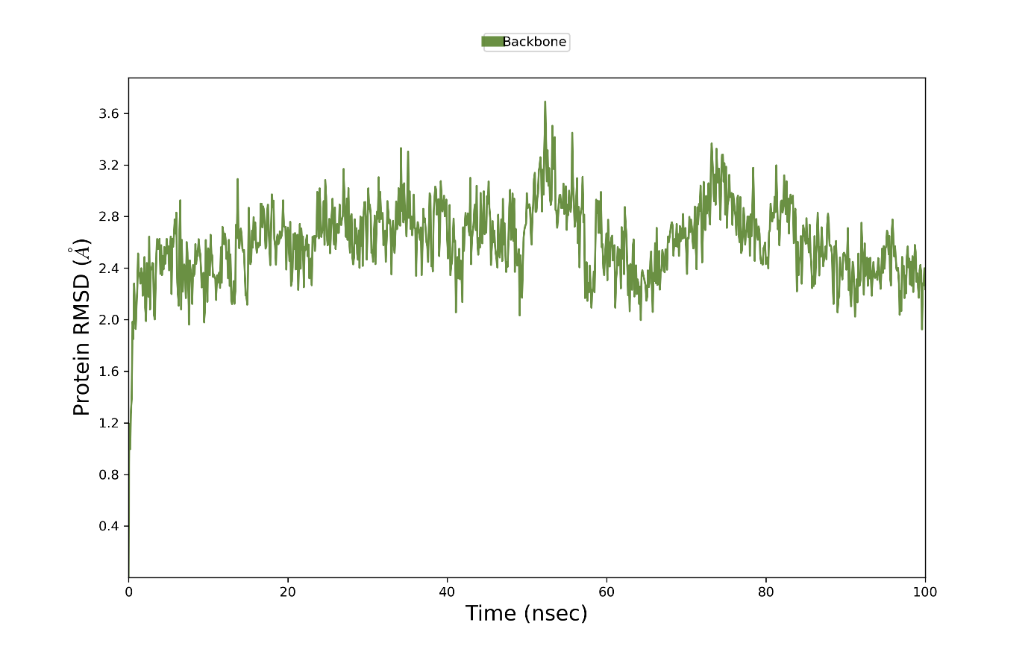


**
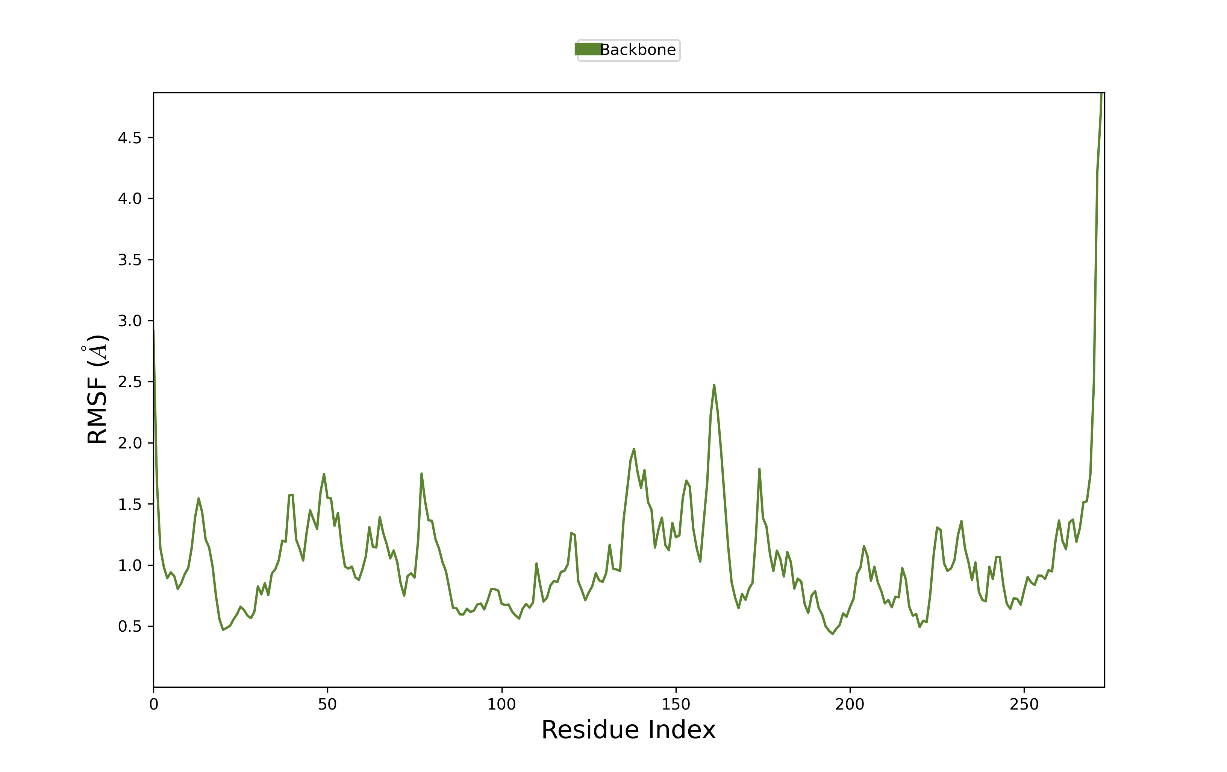
**

**Supplementary Fig 4: Molecular Simulation of SIRT5 Protein**


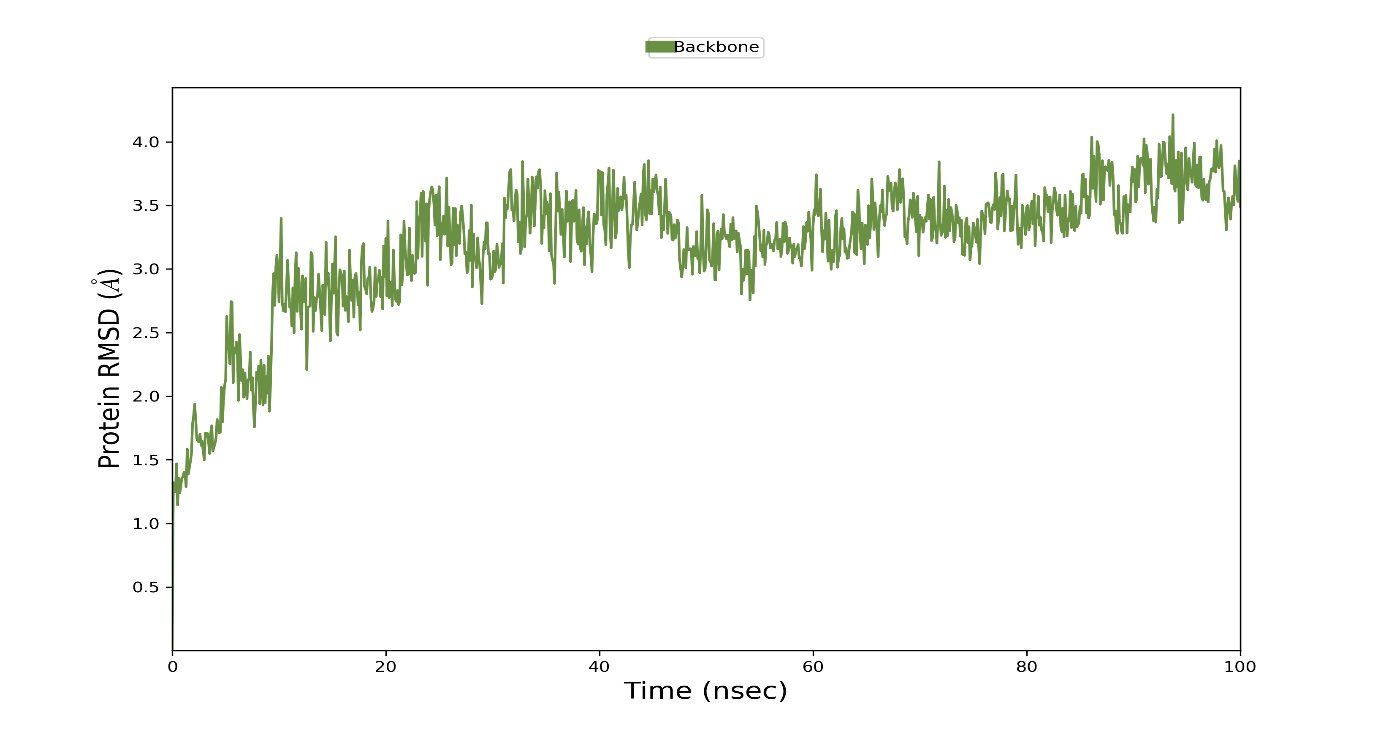


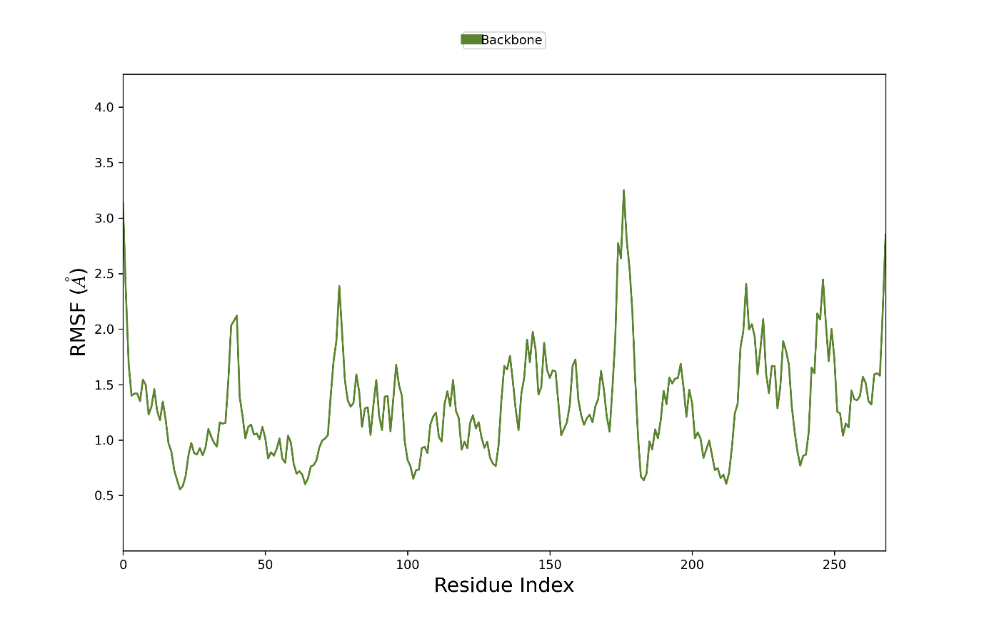


**Supplementary**
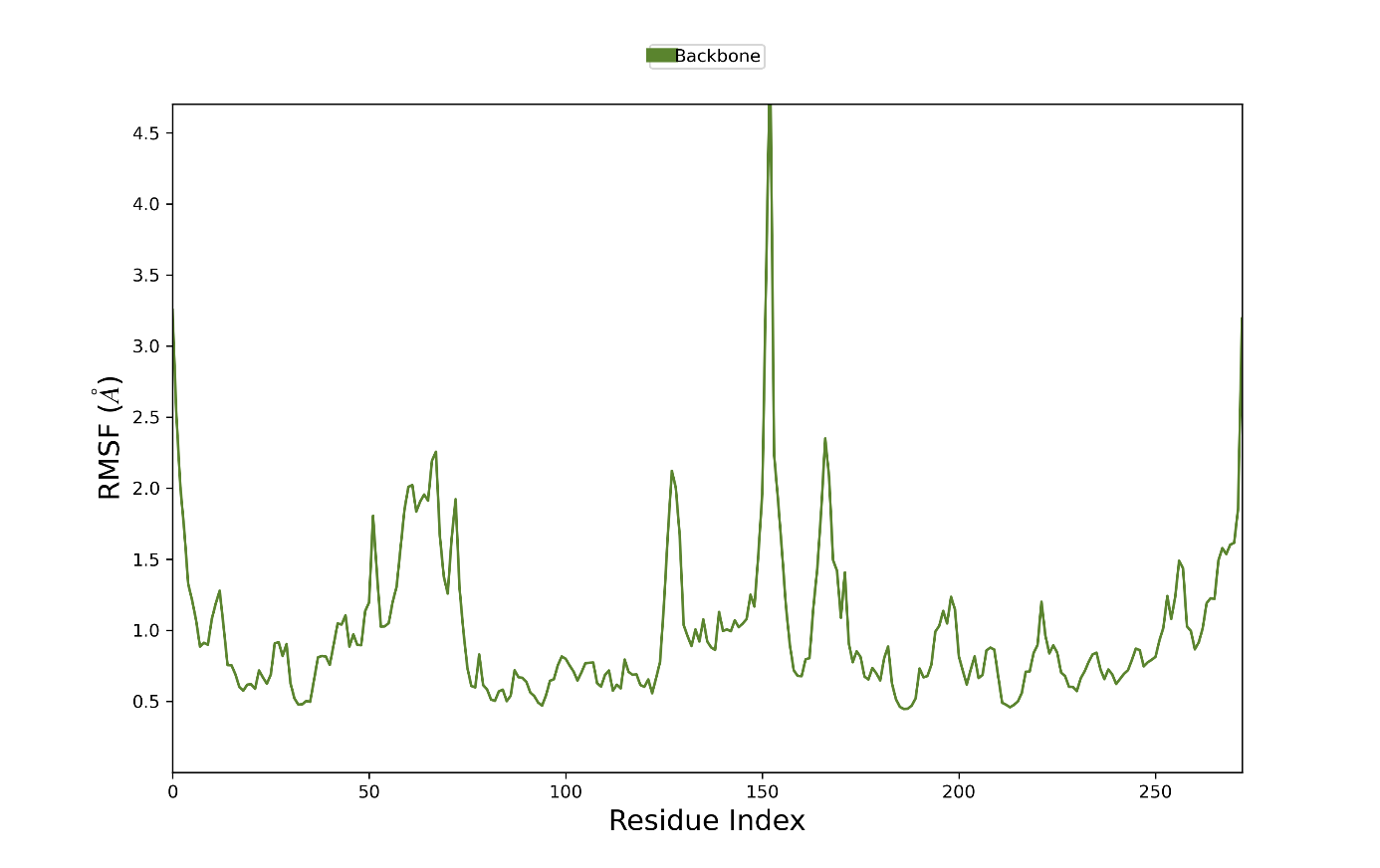
 **Fig 5: Molecular Simulation of SIRT6 Protein**


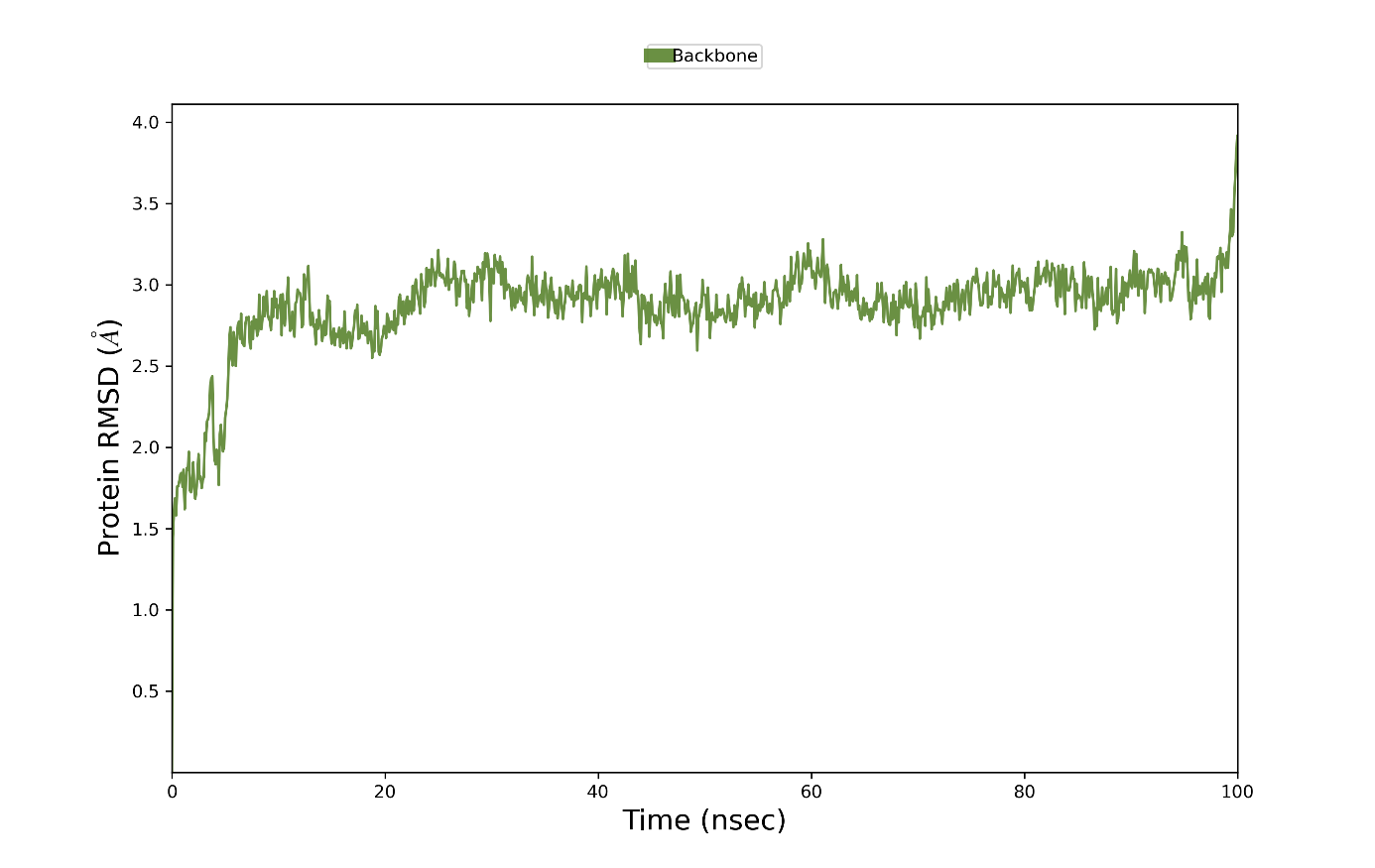


**Supplementary Fig 6: Molecular Simulation of SIRT7 Protein**


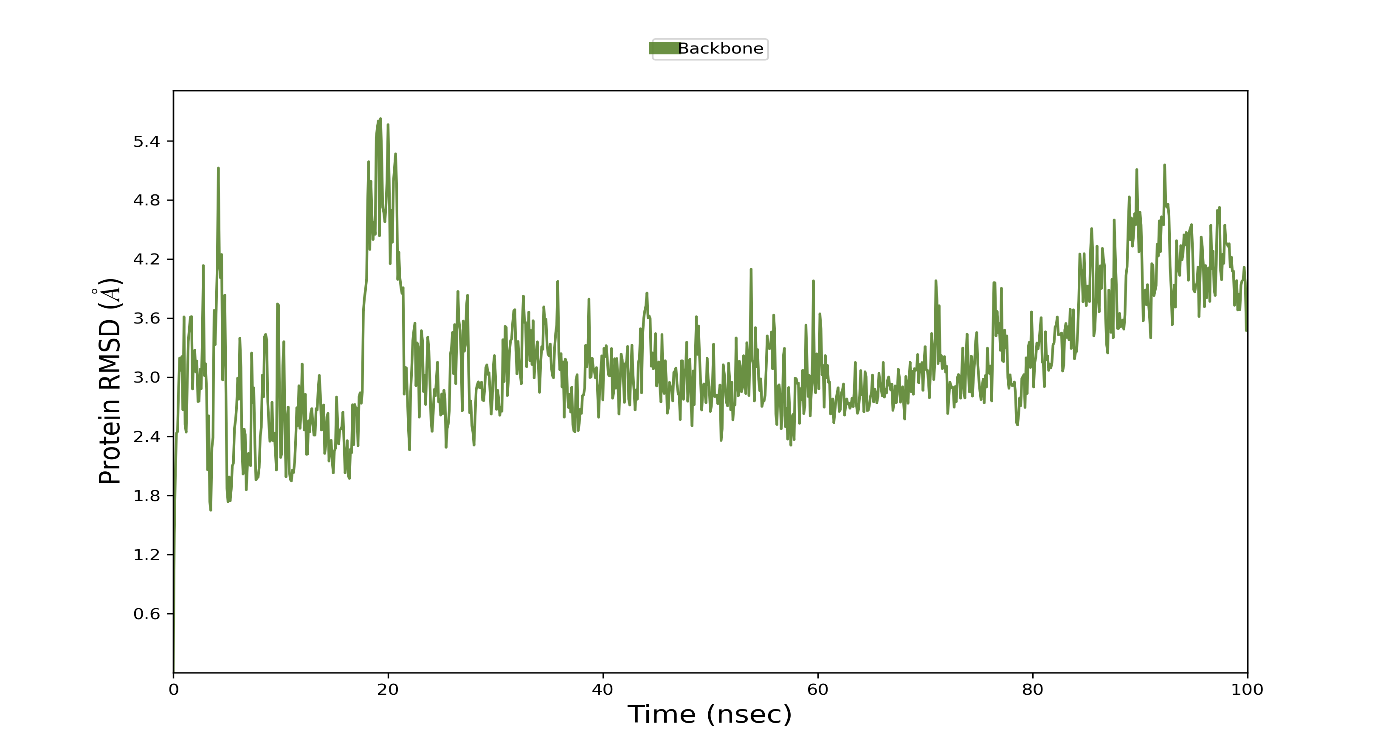


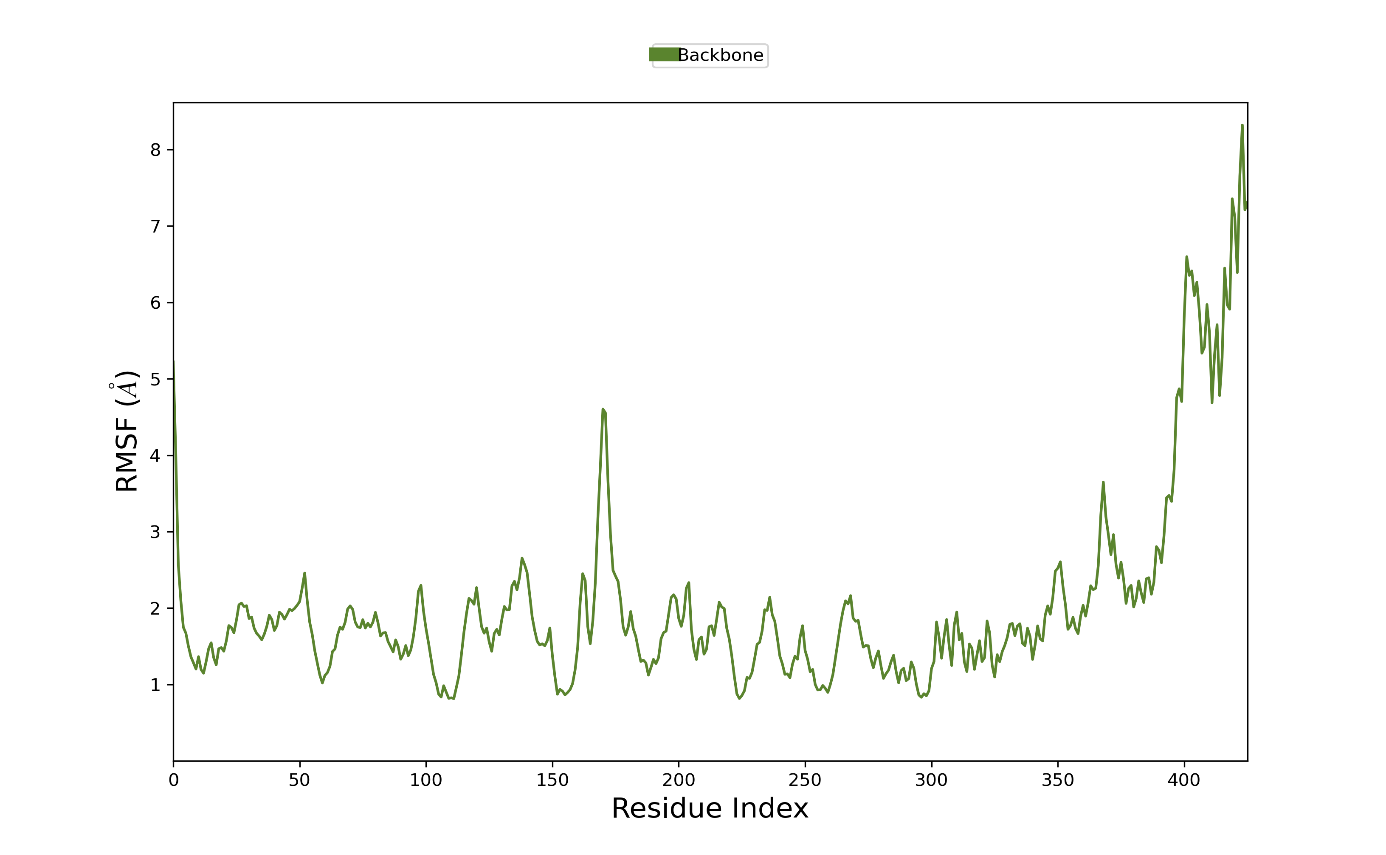

Supplement: Supplementary file 1 [file Data_Sheet_1.docx]
